# WikiPathways for plants: a community pathway curation portal and a case study in rice and arabidopsis seed development networks

**DOI:** 10.1186/1939-8433-6-14

**Published:** 2013-05-29

**Authors:** Mamatha Hanumappa, Justin Preece, Justin Elser, Denise Nemeth, Gina Bono, Kenny Wu, Pankaj Jaiswal

**Affiliations:** Department of Botany and Plant Pathology, Oregon State University, 2082 Cordley Hall, Corvallis, OR 97331-2902 USA

## Abstract

**Background:**

Next-generation sequencing and ‘omics’ platforms are used extensively in plant biology research to unravel new genomes and study their interactions with abiotic and biotic agents in the growth environment. Despite the availability of a large and growing number of genomic data sets, there are only limited resources providing highly-curated and up-to-date metabolic and regulatory networks for plant pathways.

**Results:**

Using PathVisio, a pathway editor tool associated with WikiPathways, we created a gene interaction network of 430 rice (*Oryza sativa*) genes involved in the seed development process by curating interactions reported in the published literature. We then applied an InParanoid-based homology search to these genes and used the resulting gene clusters to identify 351 *Arabidopsis thaliana* genes. Using this list of homologous genes, we constructed a seed development network in Arabidopsis by processing the gene list and the rice network through a Perl utility software called Pathway GeneSWAPPER developed by us. In order to demonstrate the utility of these networks in generating testable hypotheses and preliminary analysis prior to more in-depth downstream analysis, we used the expression viewer and statistical analysis features of PathVisio to analyze publicly-available and published microarray gene expression data sets on diurnal photoperiod response and the seed development time course to discover patterns of coexpressed genes found in the rice and Arabidopsis seed development networks. These seed development networks described herein, along with other plant pathways and networks, are freely available on the plant pathways portal at WikiPathways (http://plants.wikipathways.org).

**Conclusion:**

In collaboration with the WikiPathways project we present a community curation and analysis platform for plant biologists where registered users can freely create, edit, share and monitor pathways supported by published literature. We describe the curation and annotation of a seed development network in rice, and the projection of a similar, gene homology-based network in Arabidopsis. We also demonstrate the utility of the Pathway GeneSWAPPER (PGS) application in saving valuable time and labor when a reference network in one species compiled in GPML format is used to project a similar network in another species based on gene homology.

**Electronic supplementary material:**

The online version of this article (doi:10.1186/1939-8433-6-14) contains supplementary material, which is available to authorized users.

## Background

In many economically important plants such as cereals (rice, maize, wheat, sorghum), legumes (chickpea, soybean) and oil crops (rapeseed, oil palm), seed development is the major contributing factor towards quality and yield traits. The seed development process is dependent on growth environment conditions such as photoperiod, seasonal and diurnal rhythms, temperature fluctuations, water availability and mineral nutrition. The process is systematically coordinated by the gene products bearing functions involving metabolic enzyme activity, transport and gene expression regulation (Harmer et al. 2000; Chen et al. 2002a; Swindell et al. 2007; Hao et al. 2011). For example, late embryogenesis abundant (*LEA*) genes that are expressed during seed desiccation are also expressed in leaves undergoing dehydration (Sivamani et al. 2000). Many large and small scale studies have focused on identifying and unraveling such genetic and molecular interaction networks and on extricating developmental and regulatory pathways such as seed development in rice and legumes (Cooper et al. 2003 Le et al. 2007) and flowering time in several plant species (Putterill et al. 2004; Flowers et al. 2009; Imaizumi 2009; Michaels 2009; Higgins et al. 2010).

Plant databases like Gramene (Jaiswal 2011; Monaco et al. 2013), Kyoto Encyclopedia for Genes and Genomes (Masoudi-Nejad et al. 2007a; Masoudi-Nejad et al. 2007b), Arabidopsis Reactome (Tsesmetzis et al. 2008; D’Eustachio 2010), MetaCyc (Caspi et al. 2012) and Plant Metabolic Network (Zhang et al. 2010) are good integrated resources to study the models of metabolic pathways. The BAR (Winter et al. 2007), ARANet (Hwang et al. 2011), RiceNet (Lee et al. 2011), IntAct (Kerrien et al. 2011) and BIND (Bader et al. 2003) databases, to name a few, are excellent resources that host regulatory gene-gene interaction networks and molecular interactions. However, we also find that the pieces of information reported on existing coordinated networks are scattered across multiple online resources and are often not conveniently available to users for data analysis, editing and reintegration through community curation projects. An ideal resource for pathway data analysis is expected to empower users by providing tools for drawing, creating and editing the networks (pathways), visualization of pathways, overlaying gene expression and statistical analysis. WikiPathways, a freely available online portal, incorporates almost all of the desirable features described above, including regular updates, ease of editing and research community contributions to data management in addition to data quality administration and curation of new and existing pathways (Pico et al. 2008; Kelder et al. 2011). WikiPathways was developed as a community curation portal for pathways. It currently hosts more than 1,700 pathways from twenty-one species. These include twenty-seven pathways on its Plants Portal (http://plants.wikipathways.org) for Arabidopsis, rice and maize, making it a powerful resource for the research community and providing a central platform for pathway curation.

In this report we introduce a collaborative work on the new Plants Portal at WikiPathways developed as a community curation and analysis tool for pathway networks in plants. We use the rice (*Oryza sativa*) seed development network as an example to guide users in creating their own networks. We then show the functionality of the Pathway GeneSWAPPER (PGS) application developed by us to project a sequence homology-based seed development network for Arabidopsis (*Arabidopsis thaliana*) using rice seed development network as reference. We also present an interspecific comparison of the two networks by demonstrating the ease and functionality of visualizing gene expression data of a subset of homologous genes from the networks to compare expression at different time points during seed development while simultaneously visualizing their diurnal rhythmic expression during the seedling stage.

## Results and discussion

### Development of molecular network for rice seed development

**Figure 1:**
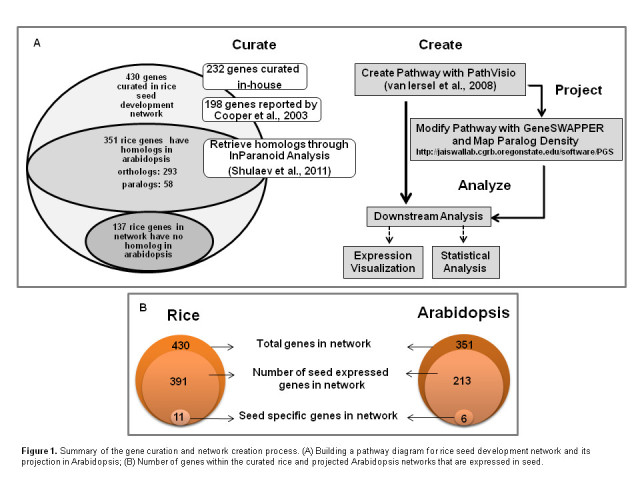
**Summary of the gene curation and network creation process.** (**A**) Building a pathway diagram for rice seed development network and its projection in Arabidopsis; (**B**) Number of genes within the curated rice and projected Arabidopsis networks that are expressed in seed.

**Figure 2:**
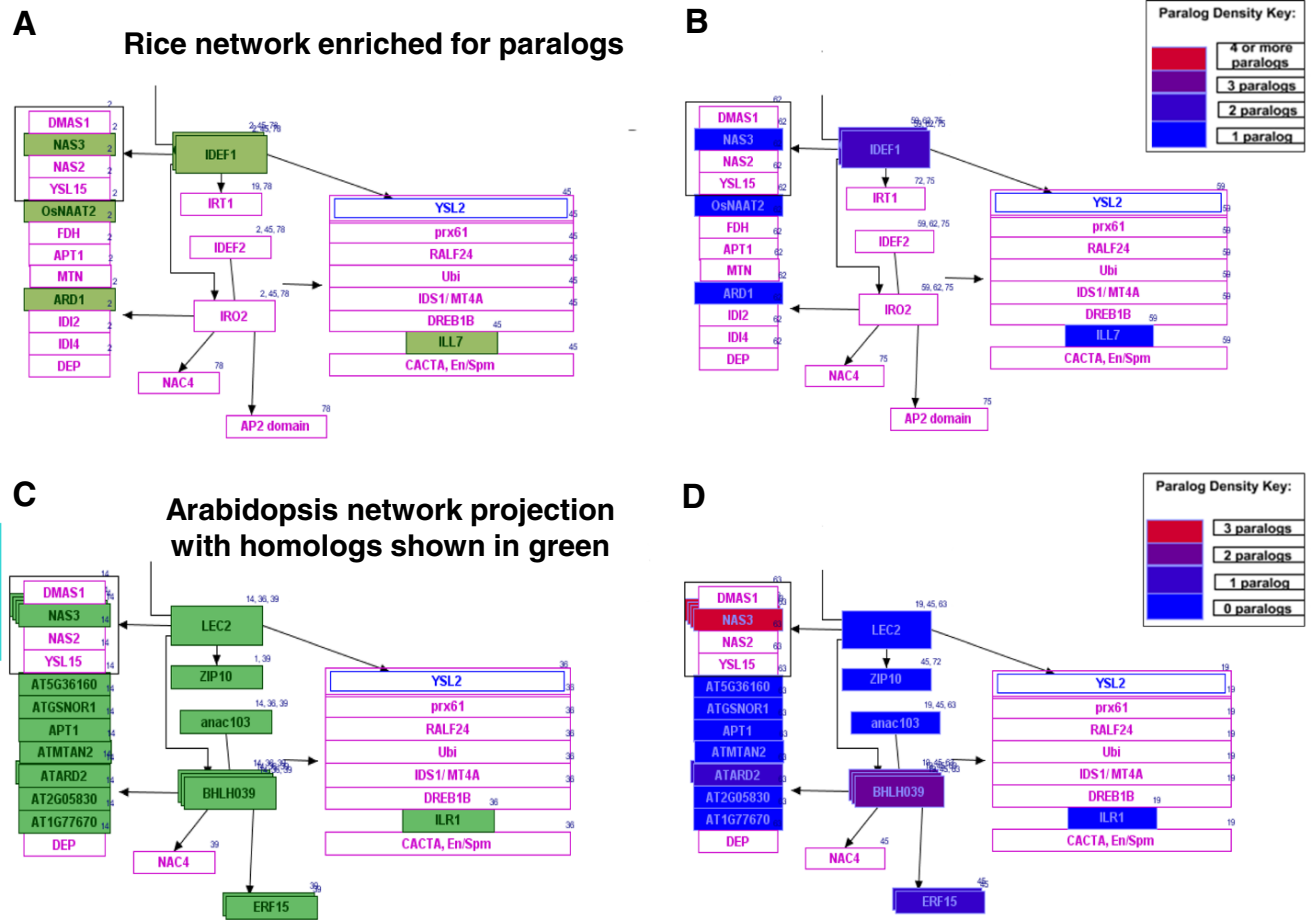
**Pathway GeneSWAPPER (PGS) can be utilized not only to project a new species network but also to enrich the existing template network.** (**A**) A section of the rice network with green colored boxes indicating rice gene paralogs; (**B**) the same section of rice network as shown in A with color highlighted boxes indicating paralog density; (**C**) a section of the Arabidopsis network showing rice gene homologs in green colored boxes; (**D**) the same section of Arabidopsis network with color highlighted boxes indicating paralog density. Figures **B** and **D** showing paralog densities displayed on the network were produced by passing the networks through Pathway GeneSWAPPER with the paralog map option enabled. The color code for number of paralogs in the images suggests blue=0/absent; purple=1; magenta=2; red=3 and/or the maximum number of paralogs in the network. However, this color scale is dynamically adjusted to depict the range between the minimum number represented by blue (1 in **B**; 0/absent in **D**) and the maximum number represented by red.

Many reports on gene expression analysis in rice seed development are available (Duan et al. 2005; Jain et al. 2007; Tian et al. 2009; Venu et al. 2011) which provide insights into co-expression and co-regulation of grain development and quality. In addition, gene interactions have also been studied extensively (Cooper et al. 2003; Kawakatsu et al. 2008; Kawakatsu et al. 2009; Mizutani et al. 2009; She et al. 2010). To predict the molecular function of a gene product it is often important to know both the expression pattern and interactions. However, a comprehensive integration of these interactions and the effect of internal and external factors such as hormones, biotic and abiotic stresses on the genes involved in seed development is lacking, beyond that presented by Cooper et al. 2003. Therefore, we developed a workflow (Figure 1) to build a reference rice seed development network (http://www.wikipathways.org/index.php/Pathway:WP2199) that includes 430 rice genes (nodes) (Additional file 1). The network includes interactions between 198 genes reported by (Cooper et al. 2003) and 232 genes and their interactions reported in various peer reviewed publications (Additional file 2). Unless shown to be different, the gene paralogs share the same interactions as the parent gene. Therefore, based on gene homology analysis we identified 109 paralogs of the 430 rice genes listed in our reference network and added them as new interactors to the reference network (Figure 2A and B, Additional file 1). The new interactors inherited the interactions of the primary gene family member identified in the reference rice network of 430 genes.

### Development of arabidopsis seed development network and comparison to rice network

We retrieved 351 homologs in Arabidopsis by querying the gene IDs of 430 curated rice genes against the gene homology datasets produced by the InParanoid-based gene family cluster analysis described under Methods. 137 rice genes returned no Arabidopsis homologs. 293 of the 351 Arabidopsis genes were true orthologs and 58 were paralogs (Additional file 1). By using the Pathway GENESWAPPER (see Methods section) and the reference rice seed development network, a gene homology-based projection of Arabidopsis seed development network was developed (http://www.wikipathways.org/index.php/Pathway:WP2279) which was then analyzed for gene loss and gain for inter- and intra-specific comparison (Figure 2). We found that the Arabidopsis gene *AtERF15* (AT2G31230) has one paralog, *AtBHLH039* (AT3G56980) has two paralogs and *AtNAS3* (AT1G09240) has three paralogs (Figure 2C and D). All of these genes have a role in iron uptake. The networks helped in identifying the rice *IRO2/* LOC_Os01g72370 gene which is a homolog of *AtBHLH039* (Figure 2A and C), *AtBHLH100* and *AtBHLH038*. The monocot grass plant species activate a chelation-based strategy II whereas dicots utilize a reduction-based strategy I (Kim and Guerinot 2007) on iron uptake. Rice, a grass species, uses both strategies but is more efficient in Fe3^+^ uptake *via* the latter method. Thus, the network projections help to build hypotheses and to ask questions about the gene family evolution, expansion/shrinkage, and the adaptation of the species with reference to its growth environment.

### Co-expression analysis of the genes in rice and Arabidopsis seed development networks

#### Gene expression profile during seed development stages

**Figure 3:**
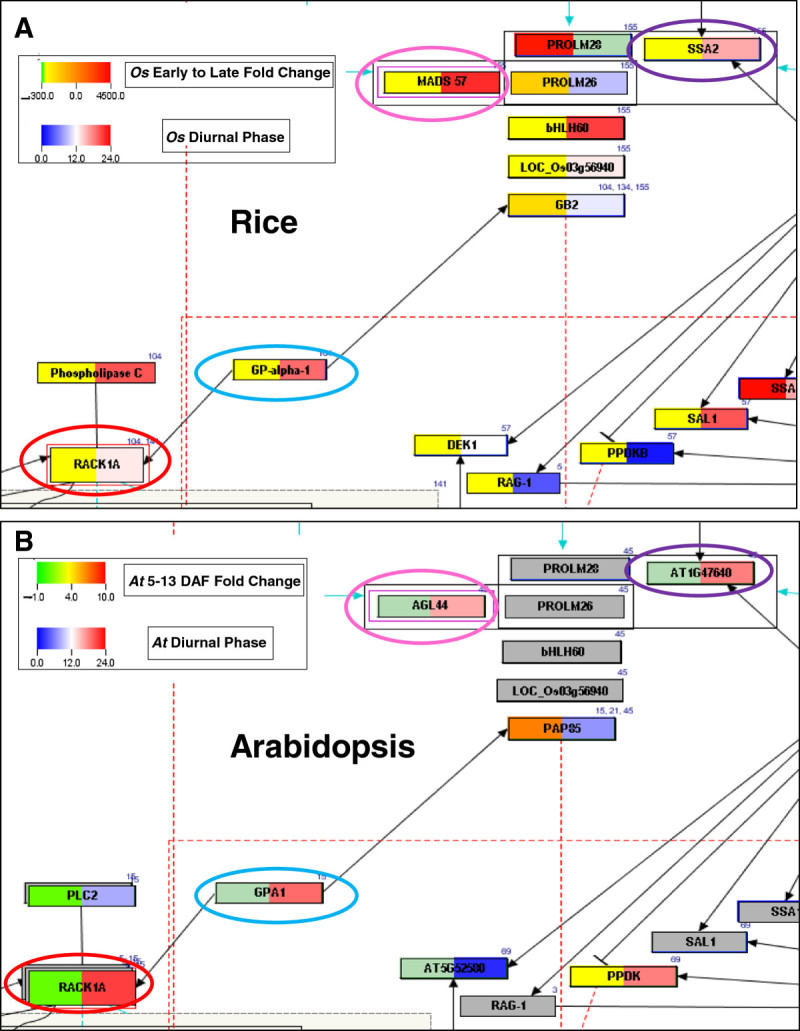
**Comparison of gene expression during seed development and diurnal rhythm in rice and Arabidopsis seed development networks.** Green-to-red color set represents expression fold changes (**A**) between 0-2DAP and 21-29DAP stages of rice seed development and (**B**) in developing Arabidopsis seed between 5-13DAF. Blue-to-red color set represents diurnal phase of expression in (**A**) rice seedlings and (**B**) Arabidopsis seedlings. Nodes that are grey in both A and B denote genes that were not queried. Nodes that are colored in the rice network but are grey in the Arabidopsis network indicate genes that lack a homolog in Arabidopsis. Panels were colored in sea green when expression values were not available. A quick visual scan of the two networks identifies similarities and differences in diurnal gene expression in the two species. For example, contrasting diurnal expression pattern is displayed by rice *RACK* 1A and its Arabidopsis homolog (circled in red), and rice *MADS57* and its homolog *AGL44* (circled in pink). However, rice *SSA2* and its homolog *AT1G47640* (circled in purple), and rice *GP-alpha-1/XA7* and its homolog *GPA1* (circled in blue) display conserved diurnal rhythmic expression.

Of the 430 rice genes in our curated reference network, 391 (~90%) were expressed in the seed, 28 genes had ‘absent’ call in the absolute analysis and 11 did not have a corresponding Affymetrix probe ID (Additional file 3). Expression of 305 of the 391 genes was up-regulated in the early stage (0–2 DAP) and expression of 86 genes was up-regulated in the late (21–29 DAP) stage of seed development (Figure 3A, Additional file 4). This was further confirmed through simple statistical calculations on PathVisio by querying (results not shown) for the number of genes with expression fold change ≤ 1 to denote those with negative fold change and down-regulation in 21–29 DAP (or up-regulation in 0–2 DAP). Eleven seed-specific genes in the rice network were identified by further comparison with data sets from Sato et al. (2011) and the gene entries listed on SeedGeneDB (http://sgdb.cbi.pku.edu.cn; Additional file 3).

A similar gene expression analysis of Arabidopsis genes in the network was performed. We identified 54 down-regulated and 28 up-regulated genes in the network (Figure 3B; Additional file 5). This was confirmed in PathVisio statistical analysis by querying for number of genes (data not shown) showing 2-fold expression change. Additionally, the Arabidopsis gene list in our projected network was compared with an earlier report (Le et al. 2010). Altogether, we identified a total of 213 (60%) Arabidopsis genes showing expression during seed development of which 6 showed seed-specific expression (Additional file 5). Though the number of seed-specific genes in our rice and Arabidopsis networks is low it does not rule out the possibility that given a different experimental condition, these and a few more genes may show expression and interactions at any given time during the plant’s development that is not restricted to seed development only.

#### Expression of rice seed storage protein coding genes during seed development

**Figure 4:**
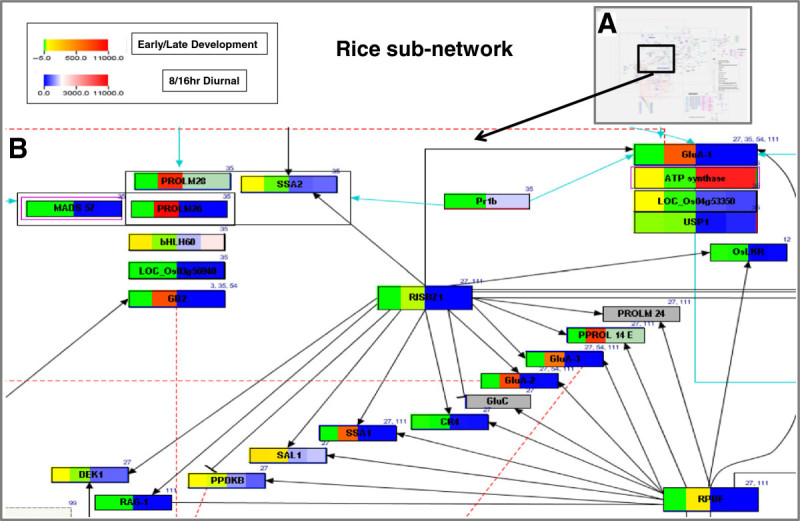
**Comparison of gene expression during seed development and diurnal rhythm for a subset of rice genes.** (**A**) Complete rice seed development network; (**B**) Expression of selected genes (microarray signal intensity average of 3 replications) in early (0-2DAP) and late (21-29DAP) stages of rice seed development (represented by color set green-to-red on panels 1 and 2 on each gene box/network node) and diurnal expression rhythm (normalized RMA values) of the same genes in rice seedlings during day (8 hr) and night (16 hr) times (represented by color set blue-to-red (panels 3 and 4 on each gene box/network node). Panels were colored in sea green when expression values were not available. Grey boxes denote genes that were not queried.

In the rice network 26 genes were found to encode for seed storage proteins of which 8 genes encoding for the globulin (LOC_Os03g57960, LOC_Os05g41970), glutelin (LOC_Os01g55690, LOC_Os10g26060, LOC_Os03g31360) and prolamin (LOC_Os05g26377, LOC_Os07g10580, LOC_Os12g16890) seed storage (nutrient reservoir) proteins were highly expressed in the seed dormancy and desiccation stage (21–29 DAP; Figure 4), and were nearly absent in the early stages of seed development (Additional file 6). These gene products are known to have some function in desiccation tolerance and may induce or enhance abiotic stress tolerance in rice and other plant species (Muench et al. 1998; Matsumura et al. 1999; Chen et al. 2002b; de los Reyes et al. 2003; Qu le et al. 2008).

#### Expression of rice MADS box genes during seed development

MADS box transcription factors function throughout plant life cycle (Alvarez-Buylla et al. 2000). During seed development they tend to express mainly in the endosperm in the early stages of development (Sato et al. 2011). All 15 MADS box proteins presented in the rice seed development network (Additional file 6) were found to express in the early globular embryo stage (0–2 DAP) except *OsMADS47* (LOC_Os03g08754) and *OsMADS57* (LOC_Os02g49840), which were absent in both early and late (21–29 DAP) stages or showed no change in expression in the late/desiccation stage (Additional file 7). *Os* MADS57 was identified under drought conditions (Cooper et al. 2003) and it is possible that it functions prior to the desiccation stage or in vegetative tissue. Of the rest, all were highly expressed in the early stage compared to post-maturation stage, indicating their importance during embryo development. Specifically, *OsMADS6* (LOC_OS02G45770) is very highly expressed in the early stage compared to the later stage (a fold change of −200) and the protein product is shown to interact directly with *OsMADS57*.

#### Diurnal gene expression during seed development

Plants keep pace with daily environmental fluctuations through the endogenous timekeeping circadian clock mechanism. Gene expression oscillates on a diurnal rhythm induced by the light/dark cycle to provide an additional regulatory gating mechanism to coordinate gene expression and function(s) with external environmental signals in promoting plant growth and development. Not surprisingly, more than one third of Arabidopsis genes are under circadian control (Pruneda-Paz and Kay 2010). Specifically, the diurnal expression pattern is critical for photosynthesis in the leaf, as seen by the number of genes expressed in mature leaf blade when compared to leaf sheath, stem and root of rice plants grown in the field (Sato et al. 2011). Further, (Sato et al. 2011) reported that most genes involved in seed development are induced quite early after flowering when endosperm cells in the embryo sac have begun accumulating starch, suggesting that the light-regulated genes may be involved in grain filling and seed maturation processes. The diurnal expression analysis of rice and Arabidopsis MADS box and seed storage protein coding genes listed in the networks revealed that the MADS box genes fall in distinct groups – some with a phase of 12 (i.e., maximum expression at 12 hours after lights on) and an end-of-day crest and another group cresting in the dark or at dawn (Additional file 6). By large, the phases of gene expression matched among the interacting genes as seen in the network diagram (Figure 3, Additional file 7). Though generally considered seed storage proteins, some prolamins are known to be expressed in seedlings also (de los Reyes et al. 2003). Therefore, we were interested in identifying genes that crest during the hot mid-day hours in combination with high expression during desiccation stage. To investigate, we queried for genes showing expression between phase-6 and −10 in statistical analyses on PathVisio. As the experiment was conducted in 12 hour photoperiods, this query is expected to coincide with 12 noon to 4 pm in natural conditions. *PROLM26* (LOC_Os07g10580) was the only gene in the subgroup to fall in this category, showing a phase-9 (Additional file 6). A closer examination showed that this gene does not display a clear rhythm, and shows a slight up-regulation at phase-9 (Additional file 8, panel B, Additional file 9, Additional file 10, panel B). Nevertheless, our analysis shows the usefulness of PathVisio in retrieving and visualizing preliminary information prior to downstream analysis.

We also observed that the diurnal rhythms of gene expression were not strictly conserved between the rice and Arabidopsis homologs. Rice is a facultative short day plant whereas Arabidopsis is a facultative long day plant. *PROML26* and *MADS57*, which are known to interact in rice, showed opposite rhythms of phase-9 and −22, respectively, in Arabidopsis. However, the diurnal rhythm pertains to seedlings where the interaction may not occur or even if it does, the gene expression rhythm may not affect the protein interaction directly. Several orthologous genes displayed contrasting rhythms, for example, *MADS3* (LOC_Os01g10504) and *AG* (AT4G18960) but genes like rice *MADS56* and its Arabidopsis ortholog *AGL19* showed very high conservation in the diurnal expression, indicating a high functional conservation as previously reported (Kater et al. 2006) (Additional file 7).

## Conclusions

In this work, we showcase the utility of PathVisio, a user-friendly, open-source software application adopted by the WikiPathways project, to build a network of genes involved in seed development in rice, an important food crop. By using publicly available microarray data sets for transcript profiles during different stages of seed development and diurnal time course we further demonstrate PathVisio’s efficacy in analysis and visualization of gene expression data sets by matching and overlaying the expression to the genes in the pathway. With the rice seed development pathway presented here, we aim to initiate and motivate community participation in the creation of pathways for many other processes in rice and other plant species. Currently, a small number of *Arabidopsis thaliana*, *Oryza sativa* and *Zea mays* pathways are represented on the Plants portal at WikiPathways. Pathways depicted for *Oryza sativa* include geranylgeranyldiphosphate biosynthesis, momilactone biosynthesis, oryzalexin S biosynthesis and the methylerythritol phosphate (MEP) pathway. The maize pathways include the most up-to-date and well-known networks for the carbon assimilation C4 pathway and carotenoid, lycopene, anthocyanin and several B-vitamin biosynthesis pathways. These pathways are a community resource hosted on the Plants portal at WikiPathways (http://plants.wikipathways.org), an open and central curation platform, where registered users can make their own contributions to plant science by creating new networks of known and/or published pathways or editing the existing ones. Registered users are cited instantly for their contribution as and when a new pathway is added and released publicly, and they are credited for any additional edits made to the existing networks and/or annotations.

We also demonstrate the Pathway GeneSWAPPER (PGS) utility application which saves valuable time and labor when a reference network from one species in the GPML format can be used to project a similar network for another species based on gene homology. The PGS paralog map feature gives users the ability to instantly identify gene families that have expanded or shrunk with respect to the number of paralogs in comparison to the reference pathway. Curation is a continuous process and additions are made to a network upon newly published experimental evidence. In addition to new projections and paralog “density” mapping, the PGS paralog map feature can be used to add new interactions to an existing pathway. On the flip side, PGS has the limitation of projecting only those genes that are represented in a reference pathway. For example, an Arabidopsis gene that lacks a homolog in rice would not be represented in the new Arabidopsis network projected using the rice network as reference. However, any additions and corrections are enabled through manual editing. Building such networks in other plant species will enhance inter-species comparison and deeper analyses through integration of already available transcript, protein and metabolomics data.

## Methods

### Network development and curation using PathVisio

PathVisio (van Iersel et al. 2008), the pathway/network editor and analysis tool of choice adopted by WikiPathways, was downloaded from the web site http://www.pathvisio.org and installed locally on the desktop. Following the instructions provided in the help documents and a set of tutorials (http://www.pathvisio.org/documentation/tutorials/), data nodes (genes and metabolites) and edges/connectors (interactions) between two nodes were drawn. The interaction arrows represent activation/up-regulation and T-bars represent inhibition/down-regulation. Groups of genes with same interactions and functional complexes were created where necessary. Each gene node is labeled with gene symbol; similarly, a metabolite node is labeled with the name of the metabolite. Subsequently, each node carries additional references to PubMed literature IDs and cites its corresponding reference database ID. For example, gene IDs refer to the Gramene Rice Ensembl/MSU6 Gene IDs and metabolites refer to either CAS numbers or ChEBI IDs depending on their availability. Any useful comments were added in free text format in the comments field. Nodes and edges/interactions were color-coded to reflect functional classification such as external stimulus, subcellular localization and self-interactions, etc.

Information on proteins involved in rice seed development and their interactions was collected from the published literature. Gene locus IDs were confirmed by mapping to MSU6 (Yuan et al. 2003) and RAP (Tanaka et al. 2008), and were represented in the rice Ensembl Gene format provided by the Gramene Database (Jaiswal et al. 2006; Youens-Clark et al. 2011). Additional information, such as experimental methods used to verify interactions, type of interaction (i.e. activation, inhibition), subcellular localization, internal and external stimulation (i.e. hormone, drought), was also collected from the published literature.

### Gene homology analysis

We carried out an InParanoid (Ostlund et al. 2010) analysis to generate gene family clusters (Shulaev et al. 2011) and thereby identify Arabidopsis genes orthologous to rice. A homolog with a score of 1.0 was considered a true ortholog and any gene with a score of ≥ 0.3 was considered a paralog in their decreasing order of homology. The list of 430 curated rice gene interactors was used to query the homology-based gene family cluster data and create a mapping file listing the gene identifiers from both species. Three gene homolog files were created (1) with rice and Arabidopsis orthologs only and (2) two separate files with rice and Arabidopsis paralogs respectively. The files include common gene symbols assigned by TAIR (http://www.arabidopsis.org) and extracted using Gramene Biomart (Spooner et al. 2012). This data is used by the Pathway GeneSWAPPER tool to either create orthology based projects for another species or enrich the existing species-specific networks by adding paralogs as new interactors.

### Pathway GeneSWAPPER (PGS) software

Beyond the benefits of curating pathway diagrams for a given species, it is also helpful to construct and visualize a similar network for another species by analyzing homologs in a graphical context. To achieve this goal, we wrote Pathway GeneSWAPPER (PGS, v0.3), a Perl software application that redraws the PathVisio pathway diagram of another species, given a reference network and gene homology (orthologs and/or paralogs) mappings. The application generates a new GPML network file containing differently colored boxes for the represented orthologs. The output of the program also includes summary information on the number of mapped and unmapped gene nodes and a compiled list of paralogs within the projected species. A configuration file allows some graphical customization (*i.e.* node color and size). As an optional feature, PGS is able to render the frequency of paralog occurrence in the projected species on a blue-to-red ‘density’ gradient – blue boxes represent a lower number of paralogs, red boxes denote a higher frequency of paralogy for that projection. PGS retains all features of the original reference network in GPML format while projecting the network onto a new species. When compared to the hours of manual intervention required to modify hundreds of network nodes by hand, this software saves time by providing a projected network from a reference template. Pathway GeneSWAPPER is freely available for download at http://jaiswallab.cgrb.oregonstate.edu/software/PGS. This website also provides links to sample data files used in this study.

### Gene expression analysis

To map the expression of genes to the subset chosen in the pathways, we downloaded the rice and Arabidopsis gene databases (Oj_Derby_20100601.bridge and At_ Derby_20100601.bridge) from the PathVisio website provided through the BridgeDb framework used for all ID mapping functions in PathVisio and WikiPathways. As required by PathVisio, the gene expression data (in CSV file format) included “Os” as system code for the Rice Ensembl Gene synonym database and “A” for the TAIR (Arabidopsis) synonym database. The expression data set was imported in PathVisio and visualization was enabled according to user-defined criteria. Statistical analysis was performed by following step-by-step instructions and criteria rules within PathVisio to pose simple but specific queries as defined in Additional files 9 and 10.

In order to investigate the seed specific gene co-expression profile of the 430 rice genes in the reference seed development network we used two gene expression data sets for rice seed development at 0–2 days and 21–29 days after pollination (DAP), available at Gene Expression Omnibus (accession GSE6893; (Jain et al. 2007)). Studying the expression of genes at the early (early globular embryo) and late developmental stages (seed maturation, dormancy and desiccation tolerance (Itoh et al. 2005)) when dormancy and desiccation are setting in, allows identification of genes that are important not only for embryo development, but also for its establishment. The publicly available data set was generated using *Oryza sativa* ssp *indica* cv IR64 grown in a culture room at 28°C under 14 hour light and 10 hour dark cycles for 15 days prior to being transferred to greenhouse. Gene expression was analyzed by the authors using GCOS 1.2.1 calculated signal intensity values averaged over 3 replications. Additional file 4 shows the number of genes up-regulated in the two stages, visualized using fold change in expression calculated from the signal intensity values (Additional file 3). Arabidopsis gene expression analysis was performed on the projected network by querying the datasets generated for transcriptome expression on 5, 9–10 and 13 days after fertilization (DAF; (Ruuska et al. 2002); Additional file 5).

For analysis of the genes expressed and regulated during diurnal conditions, we analyzed the diurnal photoperiod response data set (Mockler et al. 2007; Filichkin et al. 2011) available from the DIURNAL project website (http://diurnal.mocklerlab.org/). The publicly available dataset includes 7-day-old japonica rice (cv Nipponbare) and Arabidopsis (Col-0) seedlings that were grown in 12 hour days under 100μE (micro-Einstein) of light at 22°C day and 12°C night temperatures (LDHC; Light/Dark/Hot/Cold condition). Normalized gcRMA values were used to visualize gene expression at 8 hours after lights-on to represent day and 8 hours before lights-on (4 hours after lights-off, 16 hours after lights-on) to represent night. A phase corresponds to the respective hour after lights on.

#### Data download

The network files are available at the WikiPathways Plants Portal in GPML and BioPAX level-3 OWL formats. Network files in these formats can be imported into the Cytoscape interaction viewer. Users are suggested to first import the network in Cytoscape (Killcoyne et al. 2009) and then use its export functions to convert the network data into formats such as SBML, SIF, etc.

## Electronic supplementary material

Additional file 1:**This file has two worksheets.** (1) List of rice genes and Arabidopsis homologs in the networks. (2) List of rice paralogs. (XLSX 86 KB)

Additional file 2: List of peer reviewed references used to curate the gene interaction data and build the rice seed development network in PathVisio. (DOC 61 KB)

Additional file 3:**This file has three worksheets.** (1) Rice expressed genes and their fold change in gene expression from early to late stage of seed development (2) Input data for analyzing rice gene expression in PathVisio. Fold Change in gene expression from early to late stage of seed development in rice. (3) List of rice genes expressed in seed. (XLS 126 KB)

Additional file 4:**Visualizing transcript expression in PathVisio.** Full view of the rice seed development network showing up-regulation of rice genes in 0–2 days after pollination (DAP) (blue) and in 21–29 DAP (red). Expression data was not available for gene boxes colored in grey. (PPTX 509 KB)

Additional file 5:**This file has three worksheets.** (1) Arabidopsis seed gene expression between 5 and 13 days after fertilization (DAF). (2) List of Arabidopsis genes and their tissue/growth stage specific profile. (3) List of seed expressed Arabidopsis genes reported by Ruuska et al., 2002 and the overlap between the genes identified by our homology-based network. (XLS 884 KB)

Additional file 6: Rice and Arabidopsis gene subsets and expression values for visualization and statistical analysis. (XLS 38 KB)

Additional file 7:**Comparison of gene expression during seed development and diurnal rhythm in rice and Arabidopsis seed development networks.** Green-to-red color set represents expression fold changes (A) between 0–2 DAP and 21–29 DAP stages of rice seed development and (B) in Arabidopsis seed between 5–13 DAF. Blue-to-red color set represents diurnal phase of expression in (A) rice seedlings and (B) Arabidopsis seedlings. Nodes that are grey in both A and B denote genes that were not queried. Nodes that are colored in the rice network but are grey in the Arabidopsis network indicate genes that lack a homolog in Arabidopsis. Panels were colored in sea green when expression values were not available. A quick visual scan of the two networks identifies similarities and differences in diurnal gene expression in the two species. For example, contrasting diurnal expression pattern is displayed by rice *MADS3* and its Arabidopsis homolog *AG* (circled in red), and rice MADS57 and its homolog *AGL44* (circled in pink). However, rice *SSA2* and its homolog AT1G47640 (circled in purple), and rice *MADS56* and its homolog *AGL19* (circled in blue) display conserved diurnal rhythmic expression. (PPTX 485 KB)

Additional file 8:**Diurnal expression of some rice genes.** Arrows indicate (A) pre-dawn peak in *bHLH60* (LOC_Os08g04390); (B) small crest at mid-day (phase9) in PROLM26 (LOC_Os07g10580); (C) pre-dawn peak in *MADS57* (LOC_Os02g49840) and *MADS14* (LOC_Os03g54160); (D) end-of-day spike in *MADS5* (LOC_Os06g06750; green and magenta) and *MADS15* (LOC_Os07g01820; red and orange). The images were downloaded from the DIURNAL website (http://diurnal.mocklerlab.org/). (PPTX 158 KB)

Additional file 9:**Querying for the expression fold change between early and late seed developmental stages in a subset of rice genes using PathVisio.** (A) Results obtained after statistical analysis with user-defined criteria to identify genes with very high expression in the desiccation stage of seed development in rice. (B) Combined table showing different criteria used to query rice seed gene expression data and the results obtained. (PPTX 127 KB)

Additional file 10:**Querying for the diurnal phase of expression in a subset of rice genes using PathVisio.** (A) Number of genes that have a phase above 12 and display a diurnal crest in dark. (B) Number of genes up-regulated in the mid-day hours. (PPTX 112 KB)

Below are the links to the authors’ original submitted files for images.

Authors’ original file for figure 1

Authors’ original file for figure 2

Authors’ original file for figure 3

Authors’ original file for figure 4
