# Supplementary material for: WikiPathways for plants: a community pathway curation portal and a case study in rice and arabidopsis seed development networks
Source: Rice (N Y). 2013 May 29;6:14. doi: 10.1186/1939-8433-6-14 (PMC4883732; doi:10.1186/1939-8433-6-14)
Supplement: Supplementary file 4 — Additional file 4:Visualizing transcript expression in PathVisio. Full view of the rice seed development network showing up-regulation of rice genes in 0–2 days after pollination (DAP) (blue) and in 21–29 DAP (red). Expression data was not available for gene boxes colored in grey. (PPTX 509 KB) [file 12284_2012_51_MOESM4_ESM.pptx]

## Slide 1
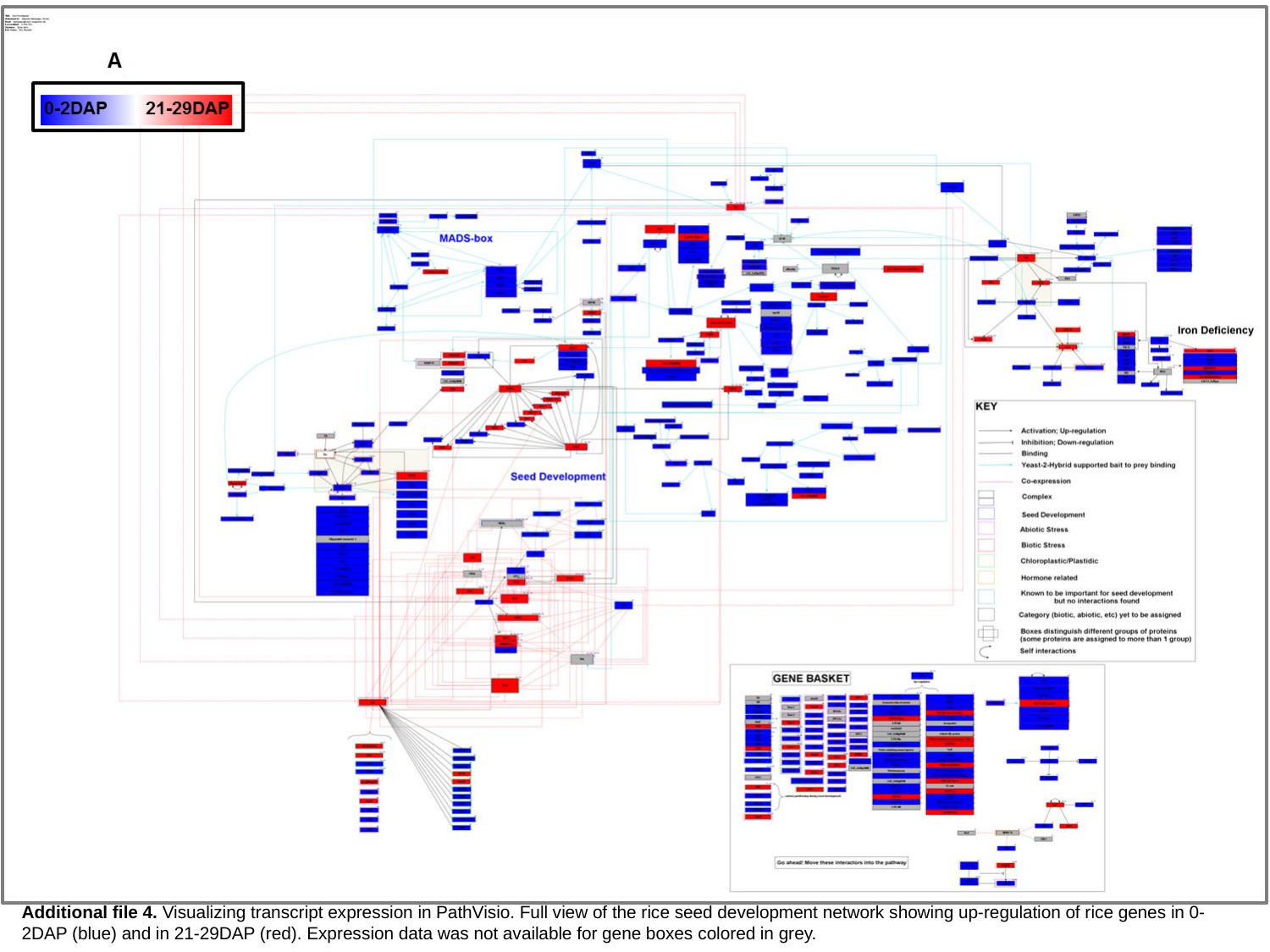

Additional file 4. Visualizing transcript expression in PathVisio. Full view of the rice seed development network showing up-regulation of rice genes in 0-2DAP (blue) and in 21-29DAP (red). Expression data was not available for gene boxes colored in grey.
