# Supplementary material for: WikiPathways for plants: a community pathway curation portal and a case study in rice and arabidopsis seed development networks
Source: Rice (N Y). 2013 May 29;6:14. doi: 10.1186/1939-8433-6-14 (PMC4883732; doi:10.1186/1939-8433-6-14)
Supplement: Supplementary file 7 — Additional file 7:Comparison of gene expression during seed development and diurnal rhythm in rice and Arabidopsis seed development networks. Green-to-red color set represents expression fold changes (A) between 0–2 DAP and 21–29 DAP stages of rice seed development and (B) in Arabidopsis seed between 5–13 DAF. Blue-to-red color set represents diurnal phase of expression in (A) rice seedlings and (B) Arabidopsis seedlings. Nodes that are grey in both A and B denote genes that were not queried. Nodes that are colored in the rice network but are grey in the Arabidopsis network indicate genes that lack a homolog in Arabidopsis. Panels were colored in sea green when expression values were not available. A quick visual scan of the two networks identifies similarities and differences in diurnal gene expression in the two species. For example, contrasting diurnal expression pattern is displayed by rice MADS3 and its Arabidopsis homolog AG (circled in red), and rice MADS57 and its homolog AGL44 (circled in pink). However, rice SSA2 and its homolog AT1G47640 (circled in purple), and rice MADS56 and its homolog AGL19 (circled in blue) display conserved diurnal rhythmic expression. (PPTX 485 KB) [file 12284_2012_51_MOESM7_ESM.pptx]

## Slide 1
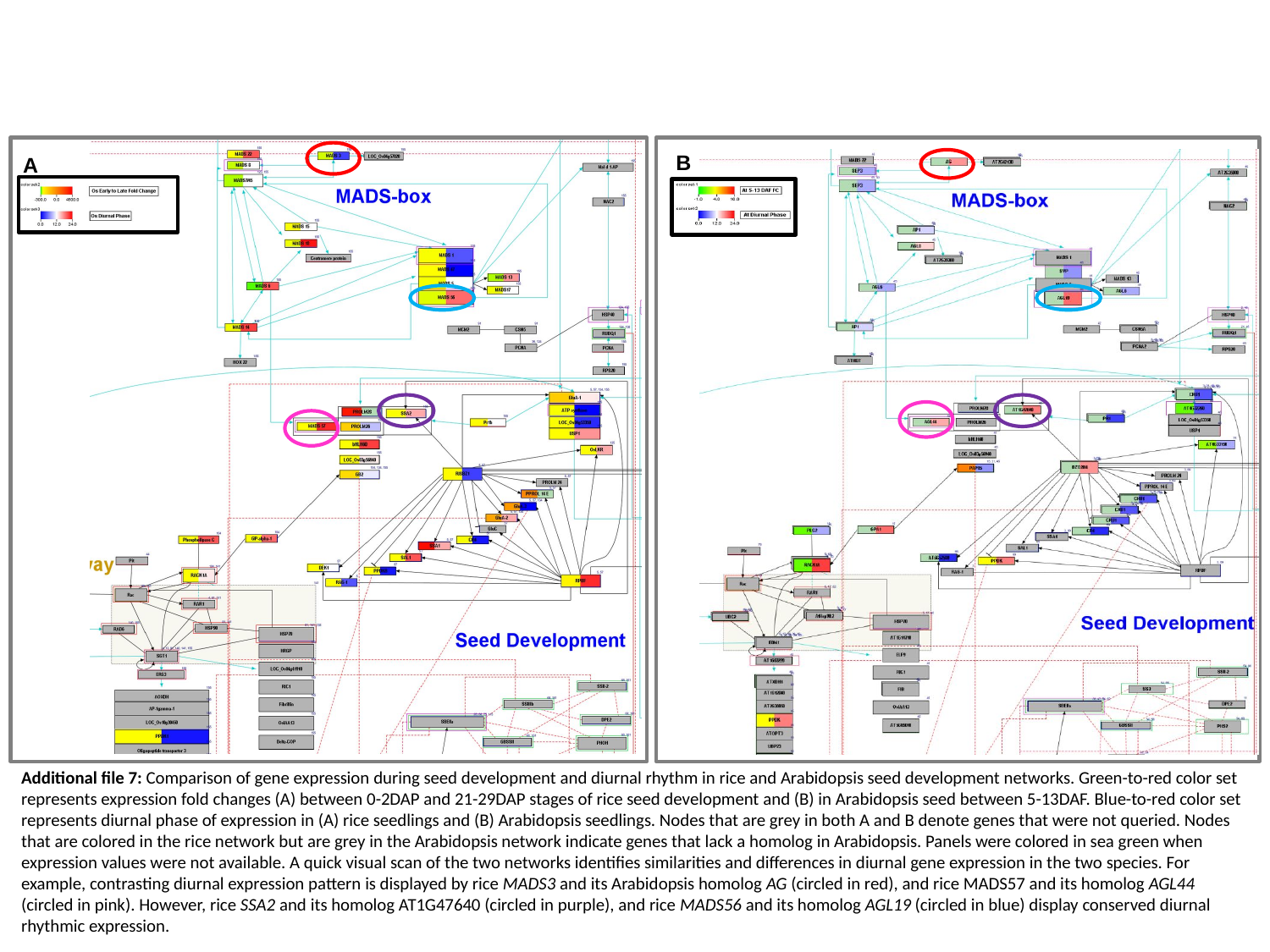

A
B
Additional file 7: Comparison of gene expression during seed development and diurnal rhythm in rice and Arabidopsis seed development networks. Green-to-red color set represents expression fold changes (A) between 0-2DAP and 21-29DAP stages of rice seed development and (B) in Arabidopsis seed between 5-13DAF. Blue-to-red color set represents diurnal phase of expression in (A) rice seedlings and (B) Arabidopsis seedlings. Nodes that are grey in both A and B denote genes that were not queried. Nodes that are colored in the rice network but are grey in the Arabidopsis network indicate genes that lack a homolog in Arabidopsis. Panels were colored in sea green when expression values were not available. A quick visual scan of the two networks identifies similarities and differences in diurnal gene expression in the two species. For example, contrasting diurnal expression pattern is displayed by rice MADS3 and its Arabidopsis homolog AG (circled in red), and rice MADS57 and its homolog AGL44 (circled in pink). However, rice SSA2 and its homolog AT1G47640 (circled in purple), and rice MADS56 and its homolog AGL19 (circled in blue) display conserved diurnal rhythmic expression.
