# Supplementary material for: WikiPathways for plants: a community pathway curation portal and a case study in rice and arabidopsis seed development networks
Source: Rice (N Y). 2013 May 29;6:14. doi: 10.1186/1939-8433-6-14 (PMC4883732; doi:10.1186/1939-8433-6-14)
Supplement: Supplementary file 8 — Additional file 8:Diurnal expression of some rice genes. Arrows indicate (A) pre-dawn peak in bHLH60 (LOC_Os08g04390); (B) small crest at mid-day (phase9) in PROLM26 (LOC_Os07g10580); (C) pre-dawn peak in MADS57 (LOC_Os02g49840) and MADS14 (LOC_Os03g54160); (D) end-of-day spike in MADS5 (LOC_Os06g06750; green and magenta) and MADS15 (LOC_Os07g01820; red and orange). The images were downloaded from the DIURNAL website (http://diurnal.mocklerlab.org/). (PPTX 158 KB) [file 12284_2012_51_MOESM8_ESM.pptx]

## Slide 1
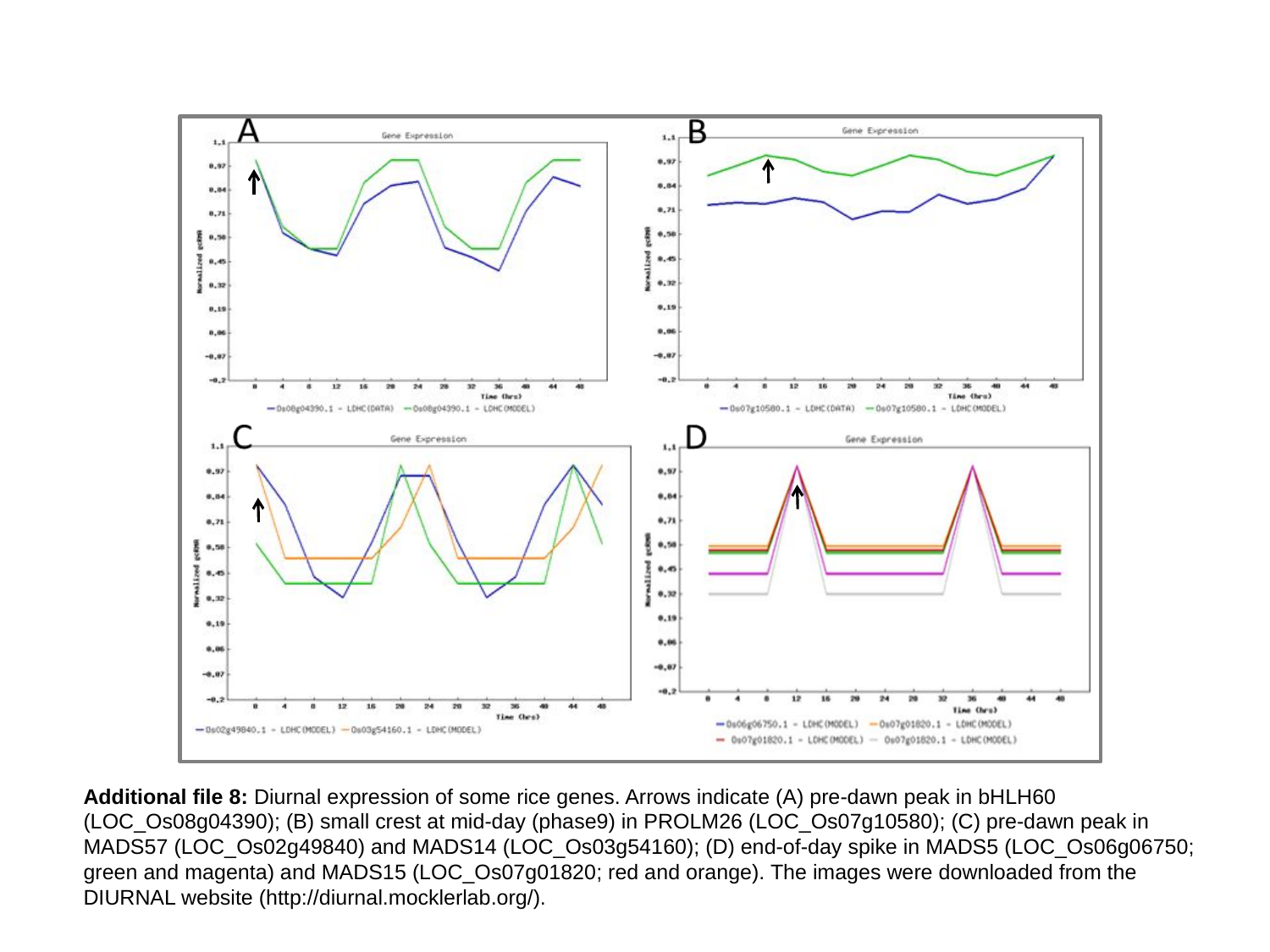

Additional file 8: Diurnal expression of some rice genes. Arrows indicate (A) pre-dawn peak in bHLH60 (LOC_Os08g04390); (B) small crest at mid-day (phase9) in PROLM26 (LOC_Os07g10580); (C) pre-dawn peak in MADS57 (LOC_Os02g49840) and MADS14 (LOC_Os03g54160); (D) end-of-day spike in MADS5 (LOC_Os06g06750; green and magenta) and MADS15 (LOC_Os07g01820; red and orange). The images were downloaded from the DIURNAL website (http://diurnal.mocklerlab.org/).
