# Supplementary material for: WikiPathways for plants: a community pathway curation portal and a case study in rice and arabidopsis seed development networks
Source: Rice (N Y). 2013 May 29;6:14. doi: 10.1186/1939-8433-6-14 (PMC4883732; doi:10.1186/1939-8433-6-14)
Supplement: Supplementary file 9 — Additional file 9:Querying for the expression fold change between early and late seed developmental stages in a subset of rice genes using PathVisio. (A) Results obtained after statistical analysis with user-defined criteria to identify genes with very high expression in the desiccation stage of seed development in rice. (B) Combined table showing different criteria used to query rice seed gene expression data and the results obtained. (PPTX 127 KB) [file 12284_2012_51_MOESM9_ESM.pptx]

## Slide 1
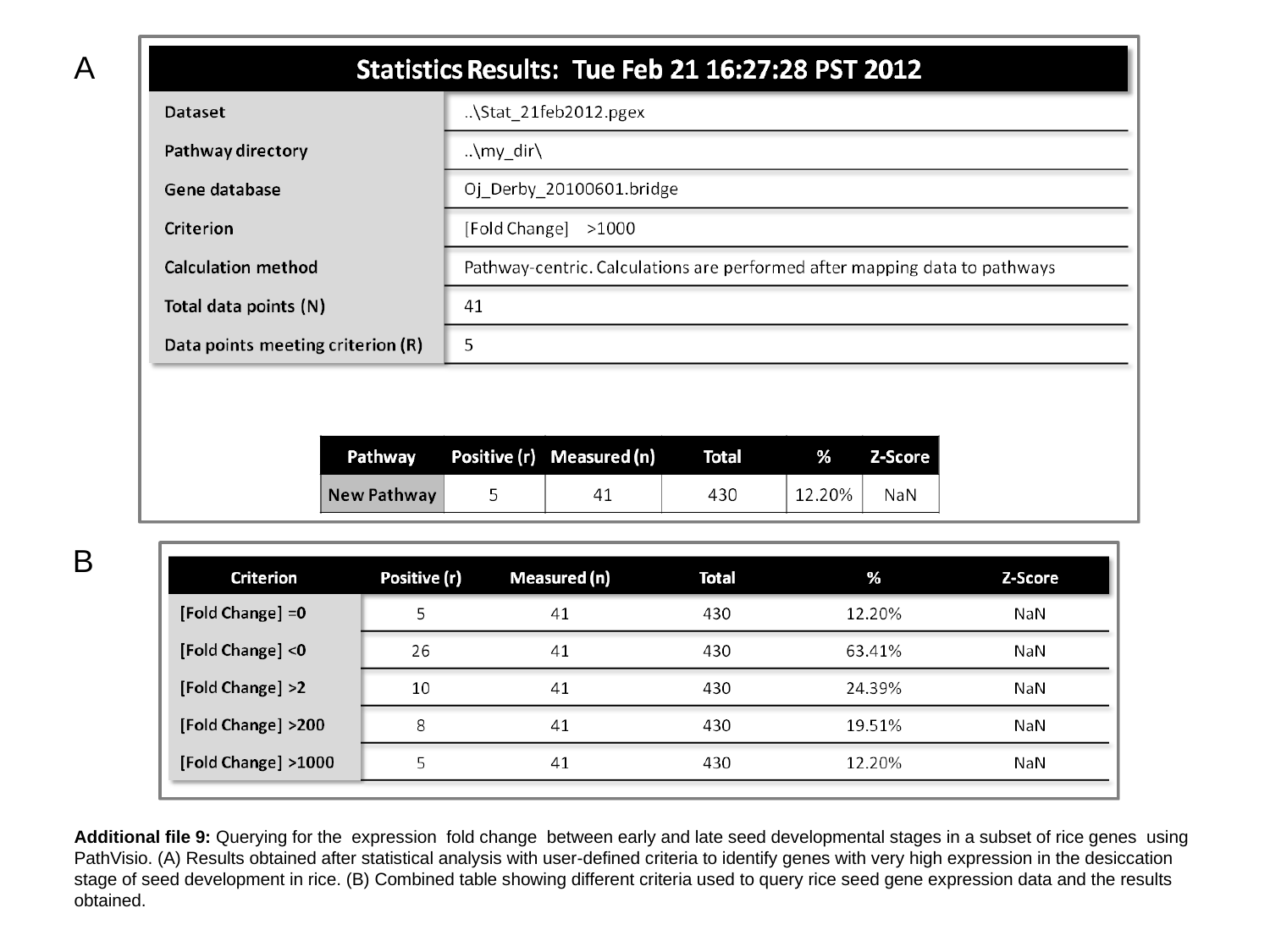

A
B
Additional file 9: Querying for the expression fold change between early and late seed developmental stages in a subset of rice genes using PathVisio. (A) Results obtained after statistical analysis with user-defined criteria to identify genes with very high expression in the desiccation stage of seed development in rice. (B) Combined table showing different criteria used to query rice seed gene expression data and the results obtained.
