# Supplementary material for: WikiPathways for plants: a community pathway curation portal and a case study in rice and arabidopsis seed development networks
Source: Rice (N Y). 2013 May 29;6:14. doi: 10.1186/1939-8433-6-14 (PMC4883732; doi:10.1186/1939-8433-6-14)
Supplement: Supplementary file 10 — Additional file 10:Querying for the diurnal phase of expression in a subset of rice genes using PathVisio. (A) Number of genes that have a phase above 12 and display a diurnal crest in dark. (B) Number of genes up-regulated in the mid-day hours. (PPTX 112 KB) [file 12284_2012_51_MOESM10_ESM.pptx]

## Slide 1
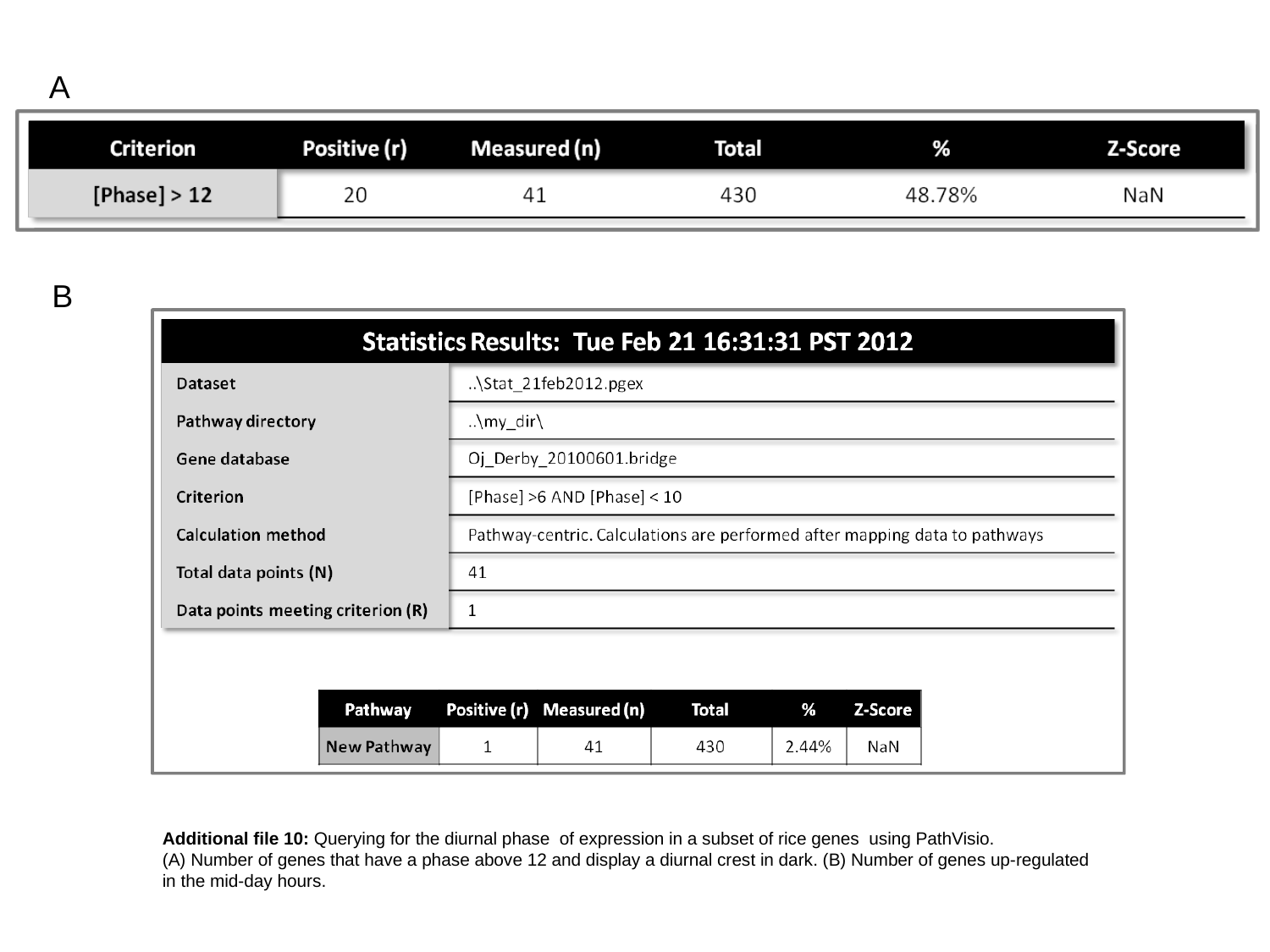

A
B
Additional file 10: Querying for the diurnal phase of expression in a subset of rice genes using PathVisio.
(A) Number of genes that have a phase above 12 and display a diurnal crest in dark. (B) Number of genes up-regulated in the mid-day hours.
