# Supplementary figures and images for: WikiPathways for plants: a community pathway curation portal and a case study in rice and arabidopsis seed development networks
Source: Rice (N Y). 2013 May 29;6:14. doi: 10.1186/1939-8433-6-14 (PMC4883732; doi:10.1186/1939-8433-6-14)

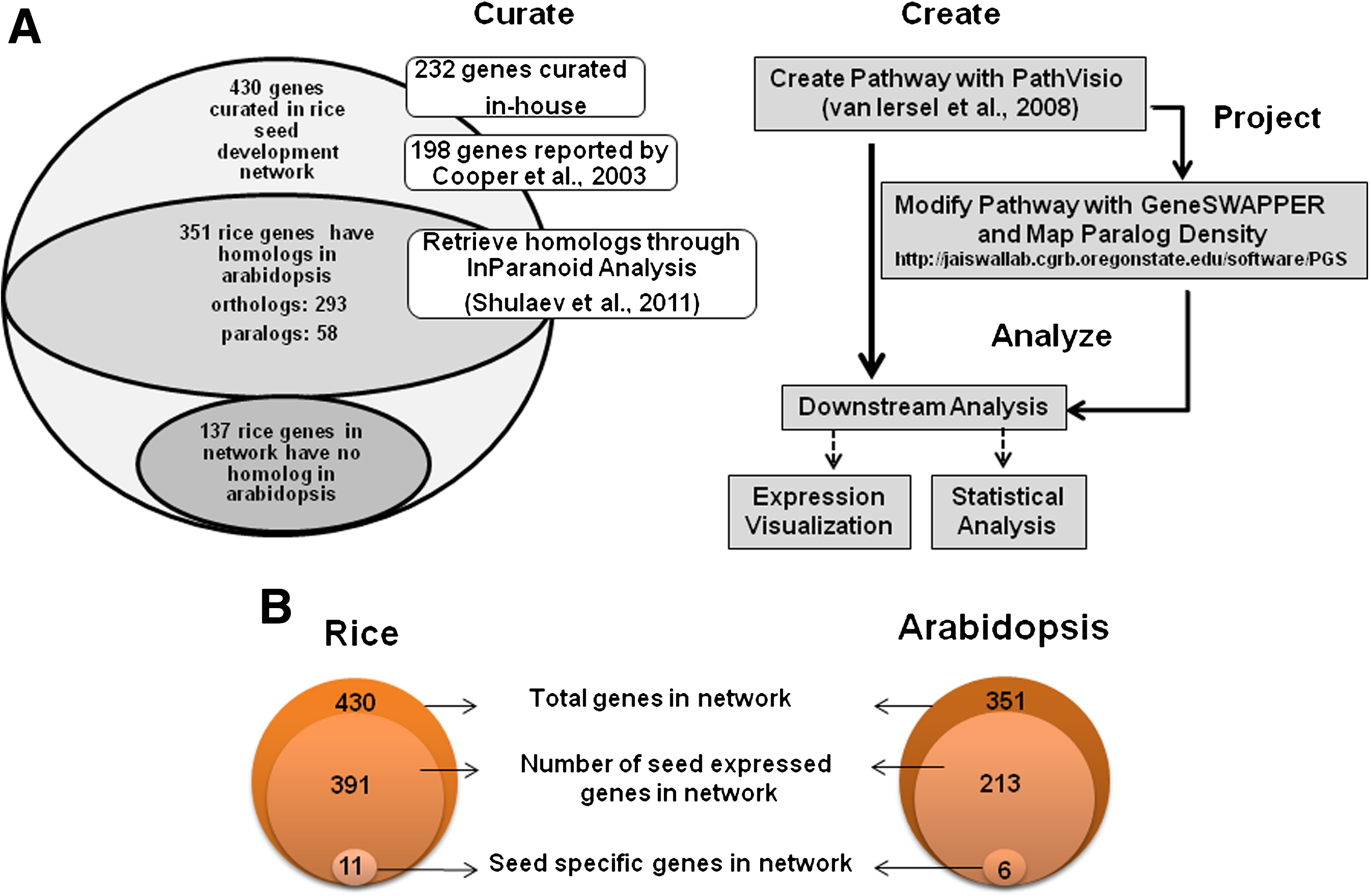

Supplement: Supplementary file 11 — Authors’ original file for figure 1 [file 12284_2012_51_MOESM11_ESM.tif]

**A**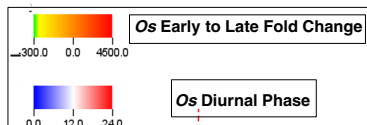**Rice**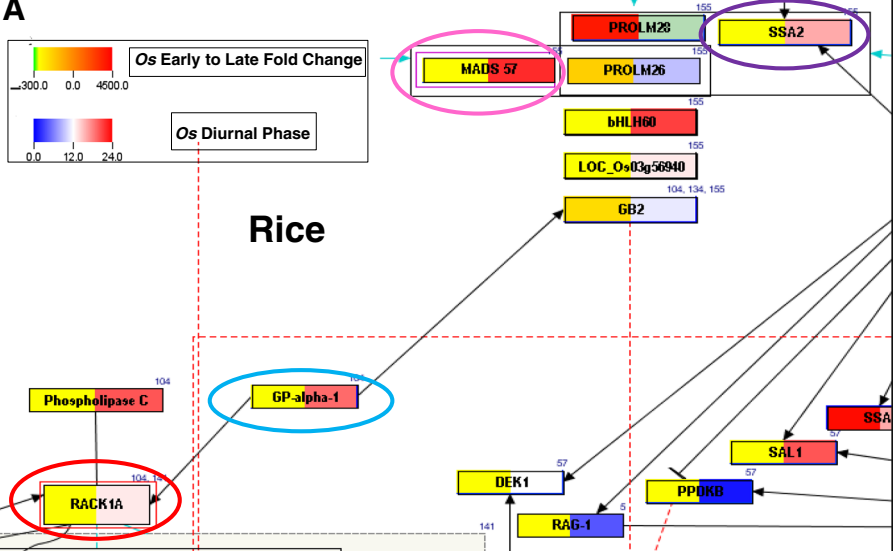**B**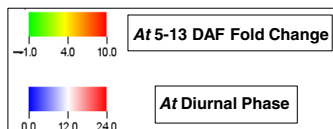**Arabidopsis**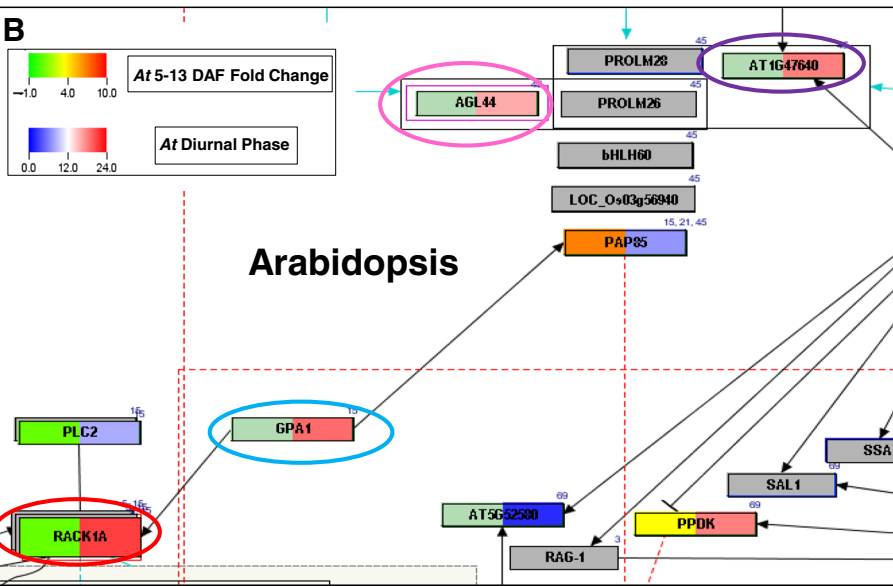

Supplement: Supplementary file 13 — Authors’ original file for figure 3 [file 12284_2012_51_MOESM13_ESM.pdf]

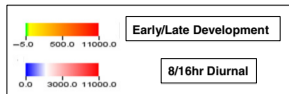

## Rice sub-network

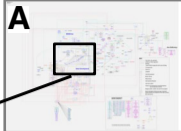

**B**

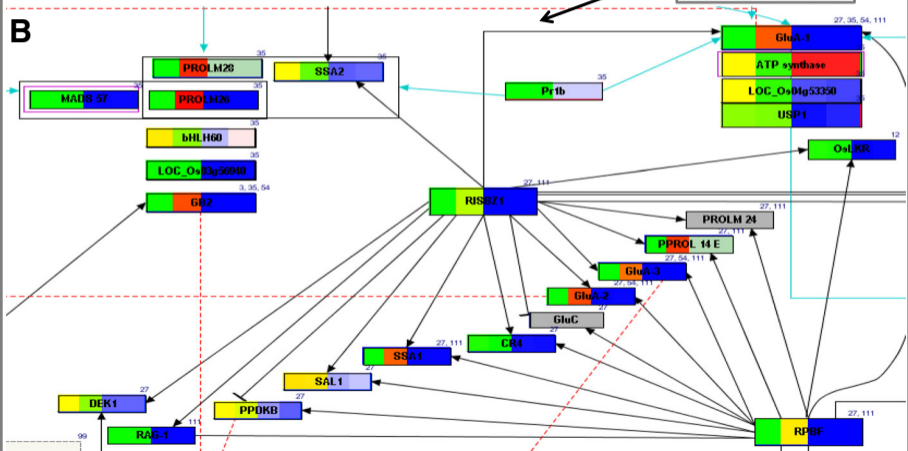

Supplement: Supplementary file 14 — Authors’ original file for figure 4 [file 12284_2012_51_MOESM14_ESM.pdf]
