# Supplementary material for: WikiPathways for plants: a community pathway curation portal and a case study in rice and arabidopsis seed development networks
Source: Rice (N Y). 2013 May 29;6:14. doi: 10.1186/1939-8433-6-14 (PMC4883732; doi:10.1186/1939-8433-6-14)
Supplement: Supplementary file 12 — Authors’ original file for figure 2 [file 12284_2012_51_MOESM12_ESM.pdf]

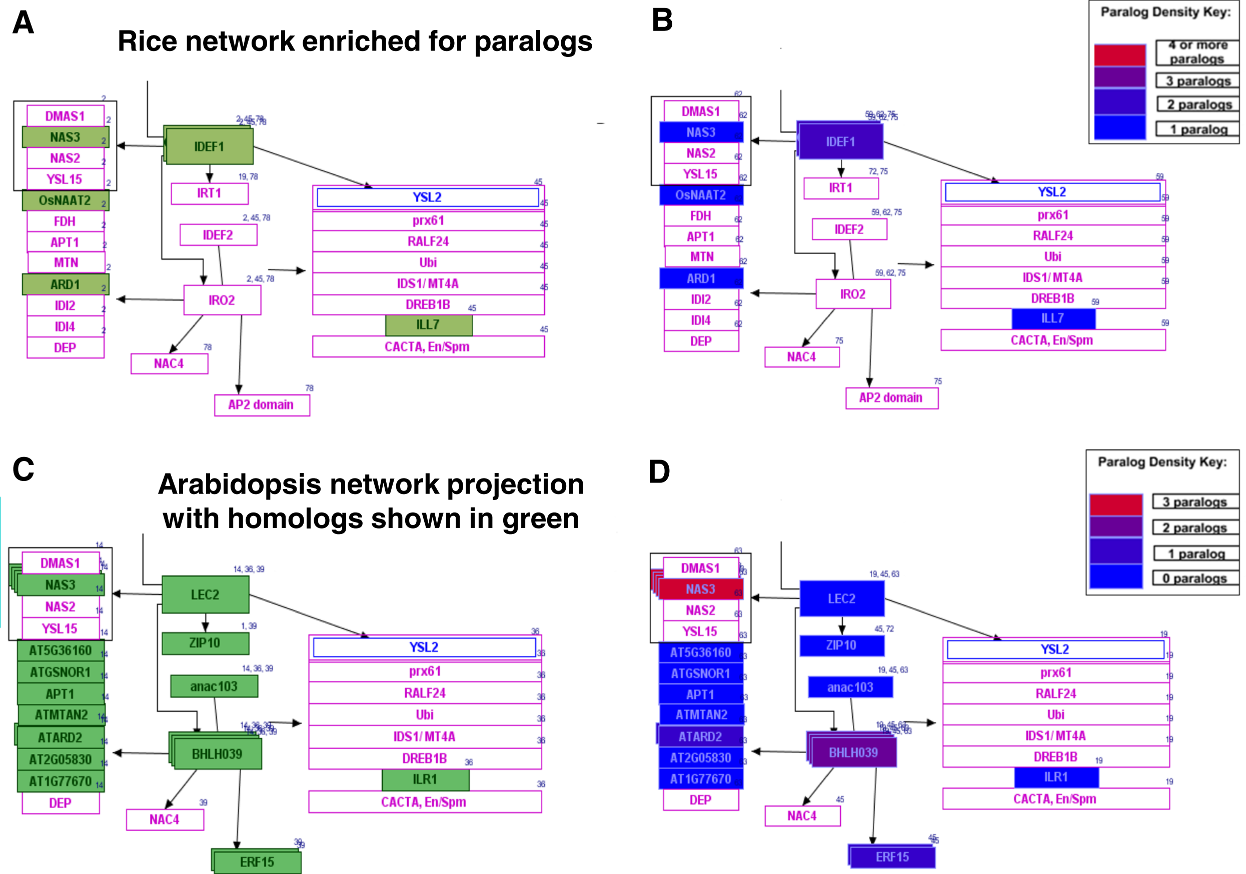

A
Rice network enriched for paralogs
DMAS1
NAS3
NAS2
YSL15
OsNAAT2
FDH
APT1
MTN
ARD1
IDI2
IDI4
DEP
IDEF1
IRT1
IDEF2
IRO2
NAC4
AP2 domain
YSL2
prx61
RALF24
Ubi
IDS1/ MT4A
DREB1B
ILL7
CACTA, En/Spm
B
Paralog Density Key:
4 or more paralogs
3 paralogs
2 paralogs
1 paralog
C
Arabidopsis network projection with homologs shown in green
LEC2
ZIP10
anac103
BHLH039
ERF15
AT5G36160
ATGSNOR1
ATMTAN2
ATARD2
AT2G05830
AT1G77670
ILR1
D
Paralog Density Key:
3 paralogs
2 paralogs
1 paralog
0 paralogs
