# Supplementary material for: WikiPathways for plants: a community pathway curation portal and a case study in rice and arabidopsis seed development networks
Source: Rice (N Y). 2013 May 29;6:14. doi: 10.1186/1939-8433-6-14 (PMC4883732; doi:10.1186/1939-8433-6-14)
Supplement: Supplementary file 2 — Additional file 2: List of peer reviewed references used to curate the gene interaction data and build the rice seed development network in PathVisio. (DOC 61 KB) [file 12284_2012_51_MOESM2_ESM.doc]

List of references used to build the rice seed development network in PathVisio.

1. Fabian T, Lorbiecke R, Umeda M, Sauter M; ''The cell cycle genes cycA1;1 and cdc2Os-3 are coordinately regulated by gibberellin in planta.; ''Planta, 2000 - [PubMed](http://www.ncbi.nlm.nih.gov/pubmed/10987556)
2. Jeong JS, Kim YS, Baek KH, Jung H, Ha SH, Do Choi Y, Kim M, Reuzeau C, Kim JK; ''Root-specific expression of OsNAC10 improves drought tolerance and grain yield in rice under field drought conditions.; ''Plant Physiol, 2010 - [PubMed](http://www.ncbi.nlm.nih.gov/pubmed/20335401)
3. Umemura K, Satou J, Iwata M, Uozumi N, Koga J, Kawano T, Koshiba T, Anzai H, Mitomi M; ''Contribution of salicylic acid glucosyltransferase, OsSGT1, to chemically induced disease resistance in rice plants.; ''Plant J, 2009 - [PubMed](http://www.ncbi.nlm.nih.gov/pubmed/18826428)
4. Yamamoto T, Mori Y, Ishibashi T, Uchiyama Y, Ueda T, Ando T, Hashimoto J, Kimura S, Sakaguchi K; ''Interaction between proliferating cell nuclear antigen (PCNA) and a DnaJ induced by DNA damage.; ''J Plant Res, 2005 - [PubMed](http://www.ncbi.nlm.nih.gov/pubmed/15806324)
5. Guo J, Wang F, Song J, Sun W, Zhang XS; ''The expression of Orysa;CycB1;1 is essential for endosperm formation and causes embryo enlargement in rice.; ''Planta, 2010 - [PubMed](http://www.ncbi.nlm.nih.gov/pubmed/19921249)
6. Shobbar ZS, Oane R, Gamuyao R, De Palma J, Malboobi MA, Karimzadeh G, Javaran MJ, Bennett J; ''Abscisic acid regulates gene expression in cortical fiber cells and silica cells of rice shoots.; ''New Phytol, 2008 - [PubMed](http://www.ncbi.nlm.nih.gov/pubmed/18315698)
7. Mao C, Wang S, Jia Q, Wu P; ''OsEIL1, a rice homolog of the Arabidopsis EIN3 regulates the ethylene response as a positive component.; ''Plant Mol Biol, 2006 - [PubMed](http://www.ncbi.nlm.nih.gov/pubmed/16786297)
8. Utsumi Y, Nakamura Y; ''Structural and enzymatic characterization of the isoamylase1 homo-oligomer and the isoamylase1-isoamylase2 hetero-oligomer from rice endosperm.; ''Planta, 2006 - [PubMed](http://www.ncbi.nlm.nih.gov/pubmed/16953433)
9. Nakamura Y, Utsumi Y, Sawada T, Aihara S, Utsumi C, Yoshida M, Kitamura S; ''Characterization of the reactions of starch branching enzymes from rice endosperm.; ''Plant Cell Physiol, 2010 - [PubMed](http://www.ncbi.nlm.nih.gov/pubmed/20305271)
10. Mao H, Sun S, Yao J, Wang C, Yu S, Xu C, Li X, Zhang Q; ''Linking differential domain functions of the GS3 protein to natural variation of grain size in rice.; ''Proc Natl Acad Sci U S A, 2010 - [PubMed](http://www.ncbi.nlm.nih.gov/pubmed/20974950)
11. Ryoo N, Yu C, Park CS, Baik MY, Park IM, Cho MH, Bhoo SH, An G, Hahn TR, Jeon JS; ''Knockout of a starch synthase gene OsSSIIIa/Flo5 causes white-core floury endosperm in rice (Oryza sativa L.).; ''Plant Cell Rep, 2007 - [PubMed](http://www.ncbi.nlm.nih.gov/pubmed/17297616)
12. Zeng D, Yan M, Wang Y, Liu X, Qian Q, Li J; ''Du1, encoding a novel Prp1 protein, regulates starch biosynthesis through affecting the splicing of Wxb pre-mRNAs in rice (Oryza sativa L.).; ''Plant Mol Biol, 2007 - [PubMed](http://www.ncbi.nlm.nih.gov/pubmed/17579813)
13. Wang Y, Gao M, Li Q, Wang L, Wang J, Jeon JS, Qu N, Zhang Y, He Z; ''OsRAR1 and OsSGT1 physically interact and function in rice basal disease resistance.; ''Mol Plant Microbe Interact, 2008 - [PubMed](http://www.ncbi.nlm.nih.gov/pubmed/18257679)
14. Schultz TF, Medina J, Hill A, Quatrano RS; ''14-3-3 proteins are part of an abscisic acid-VIVIPAROUS1 (VP1) response complex in the Em promoter and interact with VP1 and EmBP1.; ''Plant Cell, 1998 - [PubMed](http://www.ncbi.nlm.nih.gov/pubmed/9596641)
15. Xue W, Xing Y, Weng X, Zhao Y, Tang W, Wang L, Zhou H, Yu S, Xu C, Li X, Zhang Q; ''Natural variation in Ghd7 is an important regulator of heading date and yield potential in rice.; ''Nat Genet, 2008 - [PubMed](http://www.ncbi.nlm.nih.gov/pubmed/18454147)
16. Yu G, Olsen KM, Schaal BA; ''Molecular evolution of the endosperm starch synthesis pathway genes in rice (Oryza sativa L.) and its wild ancestor, O. rufipogon L.; ''Mol Biol Evol, 2011 - [PubMed](http://www.ncbi.nlm.nih.gov/pubmed/20829346)
17. Rzewuski G, Cornell KA, Rooney L, Bürstenbinder K, Wirtz M, Hell R, Sauter M; ''OsMTN encodes a 5'-methylthioadenosine nucleosidase that is up-regulated during submergence-induced ethylene synthesis in rice (Oryza sativa L.).; ''J Exp Bot, 2007 - [PubMed](http://www.ncbi.nlm.nih.gov/pubmed/17339651)
18. Hahn M, Walbot V; ''Effects of cold-treatment on protein synthesis and mRNA levels in rice leaves.; ''Plant Physiol, 1989 - [PubMed](http://www.ncbi.nlm.nih.gov/pubmed/16667159)
19. Mizutani M, Naganuma T, Tsutsumi K, Saitoh Y; ''The syncytium-specific expression of the Orysa;KRP3 CDK inhibitor: implication of its involvement in the cell cycle control in the rice (Oryza sativa L.) syncytial endosperm.; ''J Exp Bot, 2010 - [PubMed](http://www.ncbi.nlm.nih.gov/pubmed/19933315)
20. Wang YQ, Wei XL, Xu HL, Chai CL, Meng K, Zhai HL, Sun AJ, Peng YG, Wu B, Xiao GF, Zhu Z; ''Cell-wall invertases from rice are differentially expressed in Caryopsis during the grain filling stage.; ''J Integr Plant Biol, 2008 - [PubMed](http://www.ncbi.nlm.nih.gov/pubmed/18713381)
21. Wong HL, Sakamoto T, Kawasaki T, Umemura K, Shimamoto K; ''Down-regulation of metallothionein, a reactive oxygen scavenger, by the small GTPase OsRac1 in rice.; ''Plant Physiol, 2004 - [PubMed](http://www.ncbi.nlm.nih.gov/pubmed/15220467)
22. Asano T, Kunieda N, Omura Y, Ibe H, Kawasaki T, Takano M, Sato M, Furuhashi H, Mujin T, Takaiwa F, Wu Cy CY, Tada Y, Satozawa T, Sakamoto M, Shimada H; ''Rice SPK, a calmodulin-like domain protein kinase, is required for storage product accumulation during seed development: phosphorylation of sucrose synthase is a possible factor.; ''Plant Cell, 2002 - [PubMed](http://www.ncbi.nlm.nih.gov/pubmed/11910009)
23. Kobayashi T, Ogo Y, Itai RN, Nakanishi H, Takahashi M, Mori S, Nishizawa NK; ''The transcription factor IDEF1 regulates the response to and tolerance of iron deficiency in plants.; ''Proc Natl Acad Sci U S A, 2007 - [PubMed](http://www.ncbi.nlm.nih.gov/pubmed/18025467)
24. Yamamoto MP, Onodera Y, Touno SM, Takaiwa F; ''Synergism between RPBF Dof and RISBZ1 bZIP activators in the regulation of rice seed expression genes.; ''Plant Physiol, 2006 - [PubMed](http://www.ncbi.nlm.nih.gov/pubmed/16798940)
25. Ohdan T, Francisco PB Jr, Sawada T, Hirose T, Terao T, Satoh H, Nakamura Y; ''Expression profiling of genes involved in starch synthesis in sink and source organs of rice.; ''J Exp Bot, 2005 - [PubMed](http://www.ncbi.nlm.nih.gov/pubmed/16275672)
26. Cao Y, Song F, Goodman RM, Zheng Z; ''Molecular characterization of four rice genes encoding ethylene-responsive transcriptional factors and their expressions in response to biotic and abiotic stress.; ''J Plant Physiol, 2006 - [PubMed](http://www.ncbi.nlm.nih.gov/pubmed/16436304)
27. Niu X, Tang W, Huang W, Ren G, Wang Q, Luo D, Xiao Y, Yang S, Wang F, Lu BR, Gao F, Lu T, Liu Y; ''RNAi-directed downregulation of OsBADH2 results in aroma (2-acetyl-1-pyrroline) production in rice (Oryza sativa L.).; ''BMC Plant Biol, 2008 - [PubMed](http://www.ncbi.nlm.nih.gov/pubmed/18840300)
28. Cho JI, Lee SK, Ko S, Kim HK, Jun SH, Lee YH, Bhoo SH, Lee KW, An G, Hahn TR, Jeon JS; ''Molecular cloning and expression analysis of the cell-wall invertase gene family in rice (Oryza sativa L.).; ''Plant Cell Rep, 2005 - [PubMed](http://www.ncbi.nlm.nih.gov/pubmed/15759120)
29. Yamakawa H, Hirose T, Kuroda M, Yamaguchi T; ''Comprehensive expression profiling of rice grain filling-related genes under high temperature using DNA microarray.; ''Plant Physiol, 2007 - [PubMed](http://www.ncbi.nlm.nih.gov/pubmed/17384160)
30. Kobayashi T, Itai RN, Ogo Y, Kakei Y, Nakanishi H, Takahashi M, Nishizawa NK; ''The rice transcription factor IDEF1 is essential for the early response to iron deficiency, and induces vegetative expression of late embryogenesis abundant genes.; ''Plant J, 2009 - [PubMed](http://www.ncbi.nlm.nih.gov/pubmed/19737364)
31. Xie Z, Zhang ZL, Zou X, Yang G, Komatsu S, Shen QJ; ''Interactions of two abscisic-acid induced WRKY genes in repressing gibberellin signaling in aleurone cells.; ''Plant J, 2006 - [PubMed](http://www.ncbi.nlm.nih.gov/pubmed/16623886)
32. Weng J, Gu S, Wan X, Gao H, Guo T, Su N, Lei C, Zhang X, Cheng Z, Guo X, Wang J, Jiang L, Zhai H, Wan J; ''Isolation and initial characterization of GW5, a major QTL associated with rice grain width and weight.; ''Cell Res, 2008 - [PubMed](http://www.ncbi.nlm.nih.gov/pubmed/19015668)
33. Chen S, Yang Y, Shi W, Ji Q, He F, Zhang Z, Cheng Z, Liu X, Xu M; ''Badh2, encoding betaine aldehyde dehydrogenase, inhibits the biosynthesis of 2-acetyl-1-pyrroline, a major component in rice fragrance.; ''Plant Cell, 2008 - [PubMed](http://www.ncbi.nlm.nih.gov/pubmed/18599581)
34. Nakashima K, Tran LS, Van Nguyen D, Fujita M, Maruyama K, Todaka D, Ito Y, Hayashi N, Shinozaki K, Yamaguchi-Shinozaki K; ''Functional analysis of a NAC-type transcription factor OsNAC6 involved in abiotic and biotic stress-responsive gene expression in rice.; ''Plant J, 2007 - [PubMed](http://www.ncbi.nlm.nih.gov/pubmed/17587305)
35. Ji H, Kim SR, Kim YH, Kim H, Eun MY, Jin ID, Cha YS, Yun DW, Ahn BO, Lee MC, Lee GS, Yoon UH, Lee JS, Lee YH, Suh SC, Jiang W, Yang JI, Jin P, McCouch SR, An G, Koh HJ; ''Inactivation of the CTD phosphatase-like gene OsCPL1 enhances the development of the abscission layer and seed shattering in rice.; ''Plant J, 2010 - [PubMed](http://www.ncbi.nlm.nih.gov/pubmed/19807881)
36. Kasai K, Kanno T, Akita M, Ikejiri-Kanno Y, Wakasa K, Tozawa Y; ''Identification of three shikimate kinase genes in rice: characterization of their differential expression during panicle development and of the enzymatic activities of the encoded proteins.; ''Planta, 2005 - [PubMed](http://www.ncbi.nlm.nih.gov/pubmed/15891897)
37. Lee SK, Hwang SK, Han M, Eom JS, Kang HG, Han Y, Choi SB, Cho MH, Bhoo SH, An G, Hahn TR, Okita TW, Jeon JS; ''Identification of the ADP-glucose pyrophosphorylase isoforms essential for starch synthesis in the leaf and seed endosperm of rice (Oryza sativa L.).; ''Plant Mol Biol, 2007 - [PubMed](http://www.ncbi.nlm.nih.gov/pubmed/17406793)
38. Takasaki H, Maruyama K, Kidokoro S, Ito Y, Fujita Y, Shinozaki K, Yamaguchi-Shinozaki K, Nakashima K; ''The abiotic stress-responsive NAC-type transcription factor OsNAC5 regulates stress-inducible genes and stress tolerance in rice.; ''Mol Genet Genomics, 2010 - [PubMed](http://www.ncbi.nlm.nih.gov/pubmed/20632034)
39. Zhu Y, Cai XL, Wang ZY, Hong MM; ''An interaction between a MYC protein and an EREBP protein is involved in transcriptional regulation of the rice Wx gene.; ''J Biol Chem, 2003 - [PubMed](http://www.ncbi.nlm.nih.gov/pubmed/12947109)
40. Yamamoto T, Mori Y, Ishibashi T, Uchiyama Y, Sakaguchi N, Furukawa T, Hashimoto J, Kimura S, Sakaguchi K; ''Characterization of Rad6 from a higher plant, rice (Oryza sativa L.) and its interaction with Sgt1, a subunit of the SCF ubiquitin ligase complex.; ''Biochem Biophys Res Commun, 2004 - [PubMed](http://www.ncbi.nlm.nih.gov/pubmed/14733924)
41. Yi N, Kim YS, Jeong MH, Oh SJ, Jeong JS, Park SH, Jung H, Choi YD, Kim JK; ''Functional analysis of six drought-inducible promoters in transgenic rice plants throughout all stages of plant growth.; ''Planta, 2010 - [PubMed](http://www.ncbi.nlm.nih.gov/pubmed/20567981)
42. Hirose T, Terao T; ''A comprehensive expression analysis of the starch synthase gene family in rice (Oryza sativa L.).; ''Planta, 2004 - [PubMed](http://www.ncbi.nlm.nih.gov/pubmed/15232694)
43. Radchuk VV, Borisjuk L, Sreenivasulu N, Merx K, Mock HP, Rolletschek H, Wobus U, Weschke W; ''Spatiotemporal profiling of starch biosynthesis and degradation in the developing barley grain.; ''Plant Physiol, 2009 - [PubMed](http://www.ncbi.nlm.nih.gov/pubmed/19321714)
44. Yan HB, Pan XX, Jiang HW, Wu GJ; ''Comparison of the starch synthesis genes between maize and rice: copies, chromosome location and expression divergence.; ''Theor Appl Genet, 2009 - [PubMed](http://www.ncbi.nlm.nih.gov/pubmed/19593540)
45. Hattori T, Terada T, Hamasuna ST; ''Sequence and functional analyses of the rice gene homologous to the maize Vp1.; ''Plant Mol Biol, 1994 - [PubMed](http://www.ncbi.nlm.nih.gov/pubmed/8193305)
46. Wang E, Xu X, Zhang L, Zhang H, Lin L, Wang Q, Li Q, Ge S, Lu BR, Wang W, He Z; ''Duplication and independent selection of cell-wall invertase genes GIF1 and OsCIN1 during rice evolution and domestication.; ''BMC Evol Biol, 2010 - [PubMed](http://www.ncbi.nlm.nih.gov/pubmed/20416079)
47. Cooper B, Clarke JD, Budworth P, Kreps J, Hutchison D, Park S, Guimil S, Dunn M, Luginbühl P, Ellero C, Goff SA, Glazebrook J; ''A network of rice genes associated with stress response and seed development.; ''Proc Natl Acad Sci U S A, 2003 - [PubMed](http://www.ncbi.nlm.nih.gov/pubmed/12684538)
48. Komatsu S, Abbasi F, Kobori E, Fujisawa Y, Kato H, Iwasaki Y; ''Proteomic analysis of rice embryo: an approach for investigating Galpha protein-regulated proteins.; ''Proteomics, 2005 - [PubMed](http://www.ncbi.nlm.nih.gov/pubmed/16152656)
49. Kawasaki T, Okumura S, Kishimoto N, Shimada H, Higo K, Ichikawa N; ''RNA maturation of the rice SPK gene may involve trans-splicing.; ''Plant J, 1999 - [PubMed](http://www.ncbi.nlm.nih.gov/pubmed/10417713)
50. Yi N, Oh SJ, Kim YS, Jang HJ, Park SH, Jeong JS, Song SI, Choi YD, Kim JK; ''Analysis of the Wsi18, a stress-inducible promoter that is active in the whole grain of transgenic rice.; ''Transgenic Res, 2011 - [PubMed](http://www.ncbi.nlm.nih.gov/pubmed/20490919)
51. Li X, Duan X, Jiang H, Sun Y, Tang Y, Yuan Z, Guo J, Liang W, Chen L, Yin J, Ma H, Wang J, Zhang D; ''Genome-wide analysis of basic/helix-loop-helix transcription factor family in rice and Arabidopsis.; ''Plant Physiol, 2006 - [PubMed](http://www.ncbi.nlm.nih.gov/pubmed/16896230)
52. Shin JH, Kim SR, An G; ''Rice aldehyde dehydrogenase7 is needed for seed maturation and viability.; ''Plant Physiol, 2009 - [PubMed](http://www.ncbi.nlm.nih.gov/pubmed/19052152)
53. Takano-Kai N, Jiang H, Kubo T, Sweeney M, Matsumoto T, Kanamori H, Padhukasahasram B, Bustamante C, Yoshimura A, Doi K, McCouch S; ''Evolutionary history of GS3, a gene conferring grain length in rice.; ''Genetics, 2009 - [PubMed](http://www.ncbi.nlm.nih.gov/pubmed/19506305)
54. Yamamoto T, Kimura S, Mori Y, Uchiyama Y, Ishibashi T, Hashimoto J, Sakaguchi K; ''Interaction between proliferating cell nuclear antigen and JUN-activation-domain-binding protein 1 in the meristem of rice, Oryza sativa L.; ''Planta, 2003 - [PubMed](http://www.ncbi.nlm.nih.gov/pubmed/12783325)
55. Ogo Y, Kobayashi T, Nakanishi Itai R, Nakanishi H, Kakei Y, Takahashi M, Toki S, Mori S, Nishizawa NK; ''A novel NAC transcription factor, IDEF2, that recognizes the iron deficiency-responsive element 2 regulates the genes involved in iron homeostasis in plants.; ''J Biol Chem, 2008 - [PubMed](http://www.ncbi.nlm.nih.gov/pubmed/18308732)
56. She KC, Kusano H, Koizumi K, Yamakawa H, Hakata M, Imamura T, Fukuda M, Naito N, Tsurumaki Y, Yaeshima M, Tsuge T, Matsumoto K, Kudoh M, Itoh E, Kikuchi S, Kishimoto N, Yazaki J, Ando T, Yano M, Aoyama T, Sasaki T, Satoh H, Shimada H; ''A novel factor FLOURY ENDOSPERM2 is involved in regulation of rice grain size and starch quality.; ''Plant Cell, 2010 - [PubMed](http://www.ncbi.nlm.nih.gov/pubmed/20889913)
57. Nantel A, Quatrano RS; ''Characterization of three rice basic/leucine zipper factors, including two inhibitors of EmBP-1 DNA binding activity.; ''J Biol Chem, 1996 - [PubMed](http://www.ncbi.nlm.nih.gov/pubmed/8940135)
58. Shimada H, Koishihara H, Saito Y, Arashima Y, Furukawa T, Hayashi H; ''A rice antisense SPK transformant that lacks the accumulation of seed storage substances shows no correlation between sucrose concentration in phloem sap and demand for carbon sources in the sink organs.; ''Plant Cell Physiol, 2004 - [PubMed](http://www.ncbi.nlm.nih.gov/pubmed/15356337)
59. Xiao B, Huang Y, Tang N, Xiong L; ''Over-expression of a LEA gene in rice improves drought resistance under the field conditions.; ''Theor Appl Genet, 2007 - [PubMed](http://www.ncbi.nlm.nih.gov/pubmed/17426956)
60. Zou M, Guan Y, Ren H, Zhang F, Chen F; ''Characterization of alternative splicing products of bZIP transcription factors OsABI5.; ''Biochem Biophys Res Commun, 2007 - [PubMed](http://www.ncbi.nlm.nih.gov/pubmed/17604002)
61. Nakashima A, Chen L, Thao NP, Fujiwara M, Wong HL, Kuwano M, Umemura K, Shirasu K, Kawasaki T, Shimamoto K; ''RACK1 functions in rice innate immunity by interacting with the Rac1 immune complex.; ''Plant Cell, 2008 - [PubMed](http://www.ncbi.nlm.nih.gov/pubmed/18723578)
62. Thao NP, Chen L, Nakashima A, Hara S, Umemura K, Takahashi A, Shirasu K, Kawasaki T, Shimamoto K; ''RAR1 and HSP90 form a complex with Rac/Rop GTPase and function in innate-immune responses in rice.; ''Plant Cell, 2007 - [PubMed](http://www.ncbi.nlm.nih.gov/pubmed/18156216)
63. Zhang ZL, Xie Z, Zou X, Casaretto J, Ho TH, Shen QJ; ''A rice WRKY gene encodes a transcriptional repressor of the gibberellin signaling pathway in aleurone cells.; ''Plant Physiol, 2004 - [PubMed](http://www.ncbi.nlm.nih.gov/pubmed/15047897)
64. Ogo Y, Itai RN, Nakanishi H, Kobayashi T, Takahashi M, Mori S, Nishizawa NK; ''The rice bHLH protein OsIRO2 is an essential regulator of the genes involved in Fe uptake under Fe-deficient conditions.; ''Plant J, 2007 - [PubMed](http://www.ncbi.nlm.nih.gov/pubmed/17559517)
65. Oh SJ, Kim YS, Kwon CW, Park HK, Jeong JS, Kim JK; ''Overexpression of the transcription factor AP37 in rice improves grain yield under drought conditions.; ''Plant Physiol, 2009 - [PubMed](http://www.ncbi.nlm.nih.gov/pubmed/19429605)
66. Bi YM, Kant S, Clarke J, Gidda S, Ming F, Xu J, Rochon A, Shelp BJ, Hao L, Zhao R, Mullen RT, Zhu T, Rothstein SJ; ''Increased nitrogen-use efficiency in transgenic rice plants over-expressing a nitrogen-responsive early nodulin gene identified from rice expression profiling.; ''Plant Cell Environ, 2009 - [PubMed](http://www.ncbi.nlm.nih.gov/pubmed/19682292)
67. Sugimoto K, Takeuchi Y, Ebana K, Miyao A, Hirochika H, Hara N, Ishiyama K, Kobayashi M, Ban Y, Hattori T, Yano M; ''Molecular cloning of Sdr4, a regulator involved in seed dormancy and domestication of rice.; ''Proc Natl Acad Sci U S A, 2010 - [PubMed](http://www.ncbi.nlm.nih.gov/pubmed/20220098)
68. Wang E, Wang J, Zhu X, Hao W, Wang L, Li Q, Zhang L, He W, Lu B, Lin H, Ma H, Zhang G, He Z; ''Control of rice grain-filling and yield by a gene with a potential signature of domestication.; ''Nat Genet, 2008 - [PubMed](http://www.ncbi.nlm.nih.gov/pubmed/18820698)
69. Kawakatsu T, Yamamoto MP, Touno SM, Yasuda H, Takaiwa F; ''Compensation and interaction between RISBZ1 and RPBF during grain filling in rice.; ''Plant J, 2009 - [PubMed](http://www.ncbi.nlm.nih.gov/pubmed/19473328)
70. Ashikari M, Sakakibara H, Lin S, Yamamoto T, Takashi T, Nishimura A, Angeles ER, Qian Q, Kitano H, Matsuoka M; ''Cytokinin oxidase regulates rice grain production.; ''Science, 2005 - [PubMed](http://www.ncbi.nlm.nih.gov/pubmed/15976269)
71. Cho JH, Kim HB, Kim HS, Choi SB; ''Identification and characterization of a rice MCM2 homologue required for DNA replication.; ''BMB Rep, 2008 - [PubMed](http://www.ncbi.nlm.nih.gov/pubmed/18755073)
72. Fu FF, Xue HW; ''Coexpression analysis identifies Rice Starch Regulator1, a rice AP2/EREBP family transcription factor, as a novel rice starch biosynthesis regulator.; ''Plant Physiol, 2010 - [PubMed](http://www.ncbi.nlm.nih.gov/pubmed/20713616)
73. Chae HS, Cho YG, Park MY, Lee MC, Eun MY, Kang BG, Kim WT; ''Hormonal cross-talk between auxin and ethylene differentially regulates the expression of two members of the 1-aminocyclopropane-1-carboxylate oxidase gene family in rice (Oryza sativa L.).; ''Plant Cell Physiol, 2000 - [PubMed](http://www.ncbi.nlm.nih.gov/pubmed/10805599)
74. Kawakatsu T, Takaiwa F; ''Differences in transcriptional regulatory mechanisms functioning for free lysine content and seed storage protein accumulation in rice grain.; ''Plant Cell Physiol, 2010 - [PubMed](http://www.ncbi.nlm.nih.gov/pubmed/21037241)
75. Sun SJ, Guo SQ, Yang X, Bao YM, Tang HJ, Sun H, Huang J, Zhang HS; ''Functional analysis of a novel Cys2/His2-type zinc finger protein involved in salt tolerance in rice.; ''J Exp Bot, 2010 - [PubMed](http://www.ncbi.nlm.nih.gov/pubmed/20460361)
76. Song Y, You J, Xiong L; ''Characterization of OsIAA1 gene, a member of rice Aux/IAA family involved in auxin and brassinosteroid hormone responses and plant morphogenesis.; ''Plant Mol Biol, 2009 - [PubMed](http://www.ncbi.nlm.nih.gov/pubmed/19266169)
77. Arora R, Agarwal P, Ray S, Singh AK, Singh VP, Tyagi AK, Kapoor S; ''MADS-box gene family in rice: genome-wide identification, organization and expression profiling during reproductive development and stress.; ''BMC Genomics, 2007 - [PubMed](http://www.ncbi.nlm.nih.gov/pubmed/17640358)
78. Jain M, Khurana JP; ''Transcript profiling reveals diverse roles of auxin-responsive genes during reproductive development and abiotic stress in rice.; ''FEBS J, 2009 - [PubMed](http://www.ncbi.nlm.nih.gov/pubmed/19490115)
79. Kawano Y, Akamatsu A, Hayashi K, Housen Y, Okuda J, Yao A, Nakashima A, Takahashi H, Yoshida H, Wong HL, Kawasaki T, Shimamoto K; ''Activation of a Rac GTPase by the NLR family disease resistance protein Pit plays a critical role in rice innate immunity.; ''Cell Host Microbe, 2010 - [PubMed](http://www.ncbi.nlm.nih.gov/pubmed/20478538)
80. Kang SG, Jeong HK, Lee E, Natarajan S; ''Characterization of a lipoate-protein ligase A gene of rice (Oryza sativa L.).; ''Gene, 2007 - [PubMed](http://www.ncbi.nlm.nih.gov/pubmed/17376611)
